# Search-and-rescue in the Central Mediterranean Route does not induce migration: Predictive modeling to answer causal queries in migration research

**DOI:** 10.1038/s41598-023-38119-4

**Published:** 2023-08-03

**Authors:** Alejandra Rodríguez Sánchez, Julian Wucherpfennig, Ramona Rischke, Stefano Maria Iacus

**Affiliations:** 1 University of Potsdam, 14482 Potsdam, Germany; 2 Hertie School, Centre for International Security, 10117 Berlin, Germany; 3 German Centre for Integration and Migration Research (DeZIM), 10117 Berlin, Germany; 4 Institute for Quantitative Social Science, Harvard University, Cambridge, 02138 USA

**Keywords:** Human behaviour, Population dynamics

## Abstract

State- and private-led search-and-rescue are hypothesized to foster irregular migration (and thereby migrant fatalities) by altering the decision calculus associated with the journey. We here investigate this ‘pull factor’ claim by focusing on the Central Mediterranean route, the most frequented and deadly irregular migration route towards Europe during the past decade. Based on three intervention periods—(1) state-led *Mare Nostrum*, (2) private-led search-and-rescue, and (3) coordinated pushbacks by the Libyan Coast Guard—which correspond to substantial changes in laws, policies, and practices of search-and-rescue in the Mediterranean, we are able to test the ‘pull factor’ claim by employing an innovative machine learning method in combination with causal inference. We employ a Bayesian structural time-series model to estimate the effects of these three intervention periods on the migration flow as measured by crossing attempts (i.e., time-series aggregate counts of arrivals, pushbacks, and deaths), adjusting for various known drivers of irregular migration. We combine multiple sources of traditional and non-traditional data to build a synthetic, predicted counterfactual flow. Results show that our predictive modeling approach accurately captures the behavior of the target time-series during the various pre-intervention periods of interest. A comparison of the observed and predicted counterfactual time-series in the post-intervention periods suggest that pushback policies did affect the migration flow, but that the search-and-rescue periods did not yield a discernible difference between the observed and the predicted counterfactual number of crossing attempts. Hence we do not find support for search-and-rescue as a driver of irregular migration. In general, this modeling approach lends itself to forecasting migration flows with the goal of answering causal queries in migration research.

## Introduction

The Central Mediterranean Route (CMR)—between the Libyan, Tunisian, Maltese and Italian search-and-rescue areas—is one of the most frequented and dangerous irregular migration routes, with a high number migrants drowning while attempting to cross each year^1^. As a result, the rescue of people attempting to reach Europe through this route in unseaworthy vessels became a highly controversial issue in EU migration policy^2–4^, involving migrants, smugglers, NGO activists, policy makers, academics, and the media^5^. Despite an observed reduction in the number of arrivals after mid-2017, the critical humanitarian situation faced by prospective migrants in Libya, a key departure point via the Mediterranean Sea, remains unabated^6,7^.

We have identified three distinct intervention periods during the years 2011–2020 based on key changes in the politics of search-and-rescue. The first intervention period is marked by the onset of the *Mare Nostrum* operation—the largest state-led search-and-rescue operation in European history, which ran between October 18, 2013 and October 31, 2014. Although there had been sporadic search-and-rescue operations prior to the start of *Mare Nostrum*, these lacked the systematic, sustained, and coordinated effort that characterized *Mare Nostrum*, and migrants and smugglers were likely unaware of the limited search-and-rescue capacities that existed prior to this operation. The end of *Mare Nostrum*, in turn, marks a rapid increase in privately-led search-and-rescue by NGOs. Thus, we consider the start of the first private-led search-and-rescue—carried by Migrant Offshore Aid Station (MOAS) on August 26, 2014—as the start of our second intervention period. Over time, many other NGOs engaged in providing search-and-rescue in the area (see Supplementary Materials S1 for a complete overview of NGOs involved in search-and-rescue operations in the CMR) - an engagement that received widespread media attention. Although other EU-led operations were simultaneously in place at the time of the active engagement of NGOs (e.g., *Triton*), the operational area of these state-led operations was considerably smaller and further off the Libyan coast than what had been the case during *Mare Nostrum*^8,9^; in fact, after 2015, there is a marked decrease in the distance of rescue operations^10^. Finally, our third intervention period corresponds to the extension of the Libyan search-and-rescue maritime area and the increasing involvement of the Libyan Coast Guard (LCG) in coordinated pushbacks, taking place from January, 1st, 2017 onward. In fact, this period reflects a shift in policy focus from saving migrants in distress at sea to prioritizing law enforcement related to deterrence of irregular migration, including the increasing outsourcing of migration control and management to geographies outside the European borders^11^, up to the criminalization of NGOs which discursively has been linked to 'pull factor' claims and failing solidarity mechanisms within the EU^8^.

The 'pull factor' claim—as various official EU statements on the role of search-and-rescue have come to be known^12^—purports to explain the upsurge in the inflow of migrants reaching European shores during the years 2013-2016. This claim has been summarized in three related affirmations^9^: First, the presence of search-and-rescue boats encourages more crossing attempts, irrespective of the real danger of crossing the Mediterranean sea; second, state- and private-led search-and-rescue operations unintentionally help smugglers’ businesses by reducing the costs of their operations—in terms of the type of boats used and other equipment; and third, therefore, search-and-rescue has the unintended consequence of making the trip far more dangerous to migrants.

The claim that search-and-rescue could constitute a ‘pull factor'^5^ is likely based on the ‘push-and-pull factors' theory of migration^13,14^. ‘Push-and-pull factors' are structural, societal features that provide incentives for people to migrate either into or out of a given region^15^. In that sense, lack of livelihood or employment opportunities, poverty, violent and extended conflicts, corruption, and environmental degradation, linked to climate change, have been signaled out as the most salient root causes or 'push factors' of irregular migration^16^. ‘Pull factors' on the other hand refer to the better employment prospects –in terms of the availability of jobs and their associated economic opportunities, welfare benefits, safety, and respect for human rights, etc., that migrants expect to find in countries of destination in comparison to the living conditions they face in countries of origin, residence, or transit^15^. Therefore, in a strict sense, search-and-rescue cannot constitute a further ‘pull factor' within this theoretical framework. However, other theoretical frameworks centered on micro-level behavior and understandings of search-and-rescue as an institutional factor—enhancing an existing migration route—could encompass search-and-rescue as reducing the risk of crossing and, hence, inducing or sustaining a given migration flow^17^—ultimately, an issue that requires empirical investigation.

More generally, transportation systems are another factor that may ease or complicate mobility between regions. Population movements between any two regions are structurally conditioned by the presence or absence of an adequate transportation system^18^ –such as lack of roads, presence of airports, factors which relate to regional connectivity^19^, but also human-made obstacles, including visa requirements and border closures^20^. In this sense, search-and-rescue operations can, in principle, reduce the effect of a salient *intervening obstacle*^13^ –as the Mediterranean sea is– and thereby affect mobility between North Africa and Europe. Therefore, hypothetically, and *other things being equal*, search-and-rescue operations could have a causal effect on the inflow of migrants across the CMR by reducing the riskiness of crossing a natural and human-made barrier^2^. However, this claim remains contested.

Despite the high stakes and ethical nature of the ‘pull factor' claim for search-and-rescue activities (see Table S3 in Supplementary Materials for an overview of previous studies and debate), empirical evidence in favor of the ‘pull factor' claim is is both scant and methodologically compromised^21^. Existing work ignores serial auto-correlation, trends, and seasonality in the migration flow time-series data used to assess the ‘pull factor' claim, and for that reason results are based on statistical analyses not suited for stochastic time-series processes^4,22,23^. Further, most studies have only examined statistical associations between arrivals or crossing attempts and search-and-rescue in different periods^4,9,23–26^, without a proper causal identification strategy. For example, studies compare the number of crossing attempts in periods with varying levels of search-and-rescue capacity (i.e., high or low). The results of such studies suggest that state- and private-led search-and-rescue operations do not align with increases in crossing attempts. Search-and-rescue operations generally came after increases in the migration flow were observed, rendering rescue operations the effect and not the cause of the initial triggering increase, thus compromising any causal claims.

Going beyond descriptive evidence, recent assessments of the effect of search-and-rescue seek to empirically examine the causal implications of the ‘pull factor' claim. These studies differ from previous research by assuming that the flow of migrants is the outcome of a market equilibrium process^10,22,27^, where smugglers encounter migrants willing to buy crossing services offered at specific prices (i.e., a market-based approach). This market equilibrium is the result of a complex set of ‘push-and-pull factors'  that is hard to determine. Although arrivals increased considerably during the search-and-rescue period, it is possible that the increase in flow was entirely due to other root causes of migration^27^. According to this view, search-and-rescue operations increase the crossing risk by encouraging smugglers to move from wooden boats to inexpensive, inflatable boats, ultimately increasing mortality along the CMR^22^ (see also a study employing the maritime traffic through the Suez Canal as an instrumental variable)^10^. Finally, an analysis employing Agent Based Models (ABM) was able to recover the aggregate behavior of migratory movements along the CMR during the 2016-2019 period^28^. Results suggest that deterrence policies, such as interceptions at sea, obstructed the search-and-rescue operations carried out by NGOs, which led to a higher risk of death while crossing, thus—by assumption—deterring more migrants from attempting to cross.

By contrast, in this paper, we demonstrate that the ‘pull factor' claim lacks empirical support in relation to search-and-rescue in the CMR^29^. Rather, changes in the number of crossing attempts via the CMR can be well recovered by plausibly exogenous factors. We present an innovative, data-driven causal inference empirical assessment of the ‘pull factor' claim, under the assumption that search-and-rescue may affect the difficulty of travel and communication^18^ along the CMR. We improve on previous research by employing a synthetic counterfactual approach based on Bayesian structural time-series models (BSTS)^30^. This type of modeling approach combines the predictive power of multiple and exogenous – to the interventions of interest—‘push-and-pull factors' that are predictive of the number of crossing attempts $Y_t$. Crossing attempts correspond in our study to the sum of (i) arrivals to Italy and Malta $A_t$, (ii) pushbacks to Libya and Tunisia $P_t$, and (iii) recorded migrant deaths while on transit $D_t$, such that $Y_t = A_t + P_t + D_t$.

One advantage of the BSTS model, in contrast to other alternatives, is that we are able to simultaneously model data with common trends, seasonality, non-stationarity, and, particularly, high auto-correlation, as is the case of $Y_t$, and which no other method is able to do in the time-invariant or fixed treatment setting. Other more well-known methods, such as the standard difference-in-differences or synthetic control approaches, as well as other models, are inappropriate because they assume no auto-correlation or the existence of control time series that can build up a synthetic control^31^, which we lack in our context. The flexibility of the BSTS to model various time-series characteristics simultaneously makes it an attractive option that is suited to the behavior of our target time series of crossing attempts during the period of investigation (see Fig. S2 in Supplementary Materials), and data availability. Instead of the alternative approaches, we opt for building a synthetic control group out of a combination of multiple exogenous time series that are jointly predictive of our target series.

Our selection of exogenous factors $\mathbf {X_t}$ that enter the model, and which is fully documented in Table S1 in Supplementary Materials, reflects the fact that irregular migration is the result of the complex dynamics of various economic and social systems^32^, and is based on a spikes-and-slabs prior for the selection of covariates, embedded in the BSTS^30^. Our predictive factors include various factors known to impact migration, such as, conflicts, commodity prices, unemployment rates or job search indicators, currency exchanges, and environmental disasters, that were observed during the period 2011–2020 in different geographies. We also include within our predictive factors the airport flows between countries in Africa and Europe given that these may capture further unobserved factors that could affect flow through the CMR, considering that airport flows are unlikely to be affected by our intervention periods and measure an altogether different form of mobility. Our predictive models are based on information on the association between time series from predictive features and observed crossing attempts during the pre-intervention period $t \le \tau$, where $\tau$ denotes the start of one of our three intervention periods. We use the models fitted to pre-intervention data to predict the expected number of crossing attempts $\hat{Y}_{t > \tau }$ in the post-intervention period, and subsequently compare the observed crossing attempts with our counterfactual prediction. A substantial difference between $\hat{Y}_{t > \tau }$ and $Y_{t > \tau }$ would be indicative of an effect—when positive for an increase of the flow after the intervention, and a decrease in the flow when negative—that can be attributed to the start of the intervention of interest.

## Results

### Descriptive trends

**Figure 1:**
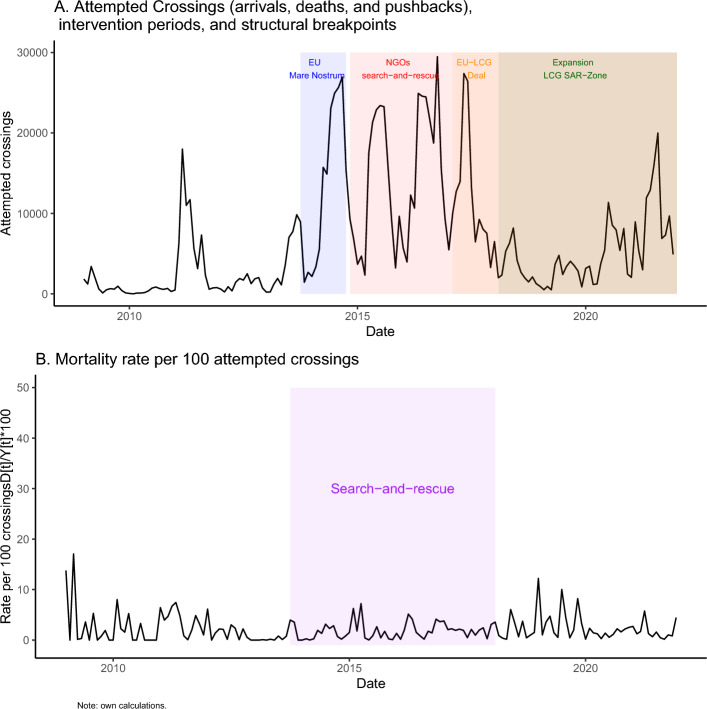
Time-series of crossing attempts, mortality rate, and main intervention periods, 2009–2021.

Figure 1 shows the number of crossing attempts over time (Panel A) alongside the mortality rate per 1000 crossing attempts (Panel B). Panel A suggest the presence of seasonality and trend components in the series—which we confirm by decomposing the time-series into trend, seasonal, and random components^33^ (see Fig. S2 in Supplementary Materials); whereas Panel B suggests substantial differences in the mortality rate over time. Panel A visually separates the distinct intervention periods we seek to evaluate. As pointed out by previous research, the state-led and private-led search-and-rescue periods in blue and red, respectively, do coincide with a higher number of crossing attempts, especially in contrast to the periods following the EU-Libya cooperation agreements. Figure 1B shows that, in contrast to some of the previous literature, during the search-and-rescue period highlighted in purple (both state-led and private-led), the mortality rate was lower and less volatile than before and after search-and-rescue periods. The preceding period, from the start of our data in 2010 until late 2013 is marked by the absence of systematic, sustained, and coordinated search-and-rescue operations; whereas the after search-and-rescue period corresponds to the extension of the Libyan search-and-rescue zone and the EU-Libya coordinated pushbacks. Importantly, search-and-rescue is not associated with a higher estimated mortality rate; if anything this rate is lower under search-and-rescue. By contrast, the absence of search-and-rescue and the start of coordinated pushbacks are associated with a higher estimated mortality rate. Due to unreported or undetected migrant deaths at sea, this estimated mortality rate should be interpreted with caution given the uncertainty in both the numerator and denominator counts used to estimate this rate^1,24^.

### Counterfactual analysis

**Figure 2:**
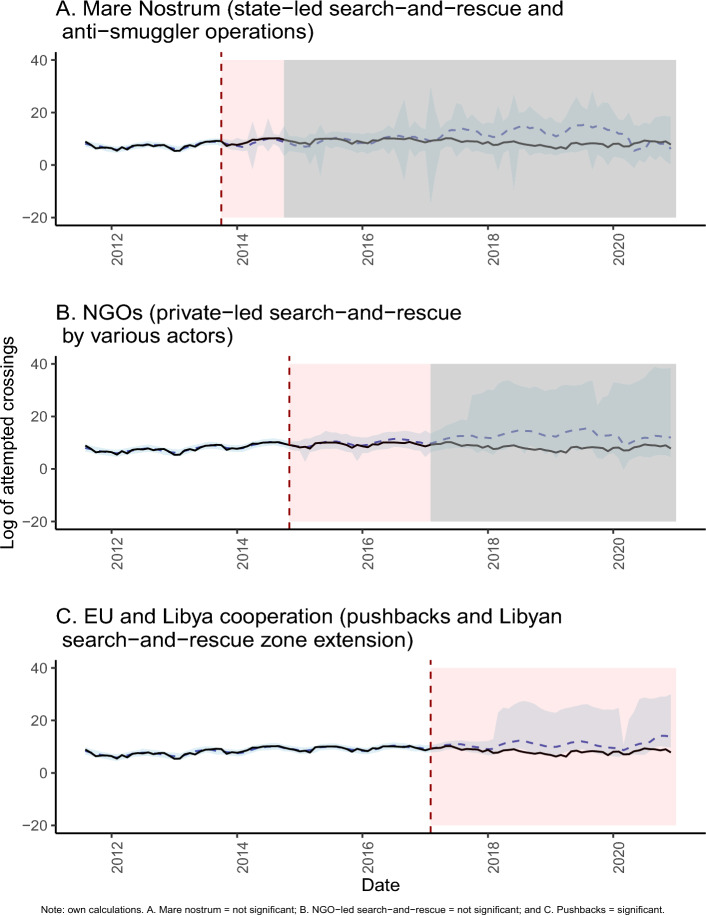
Intervention periods and the predicted counterfactual and observed time-series (log scale), 2011–2020.

Figure 2 shows the results of the predictive modeling, which further contradict the ‘pull factor' claim. Panels A, B and C show the observed time-series in a logarithmic scale in black (identical across all three panels), and the corresponding predicted counterfactual time-series with their respective 95% confidence intervals in lighter blue. Our set of selected 'push-and-pull factors' can predict the trends in crossing attempts during the three pre-intervention periods. In Supplementary materials S1 we show which factors are strongly predictive of the crossing attempts, as highlighted by the spike-and-slab variable selection models (e.g., these included some conflict intensity, some commodity prices, weather indicators, currency exchanges, some natural disasters, and the air traffic flows between countries and the EU). Importantly for our identification strategy, it is worth emphasizing that none of these predictors could have been affected by any of the interventions we are interested in (which we show in Tables S4–S6 in Supplementary Materials by means of Granger causality tests). Based on these selected covariates, we built the counterfactual crossing attempts time-series and compared it against the observed time-series. The three predicted counterfactual time-series (in blue) differ slightly between the three panels because the respective state-space models rely on a different number of time points and covariates to ’learn’ the dynamics of crossing attempts over time. The BSTS model is able to improve its predictive abilities for the later interventions - the EU and Libya agreements, which took place at a much later period in our observation window, for which more information is available. Nevertheless, our predictions closely mimic the observed time-series during all our pre-intervention periods, (i.e., the observed time-series generally falls within the narrow confidence intervals of our predicted counterfactual), capturing both trend and seasonality. This lends strong validation to our counterfactual estimate in the post-intervention period, given that a good fit in the pre-intervention period is the ultimate test of how good our model could be. In Supplementary Materials S1, we perform model validation for some of the components of the model which suggest our results are robust to other specifications.

**Figure 3:**
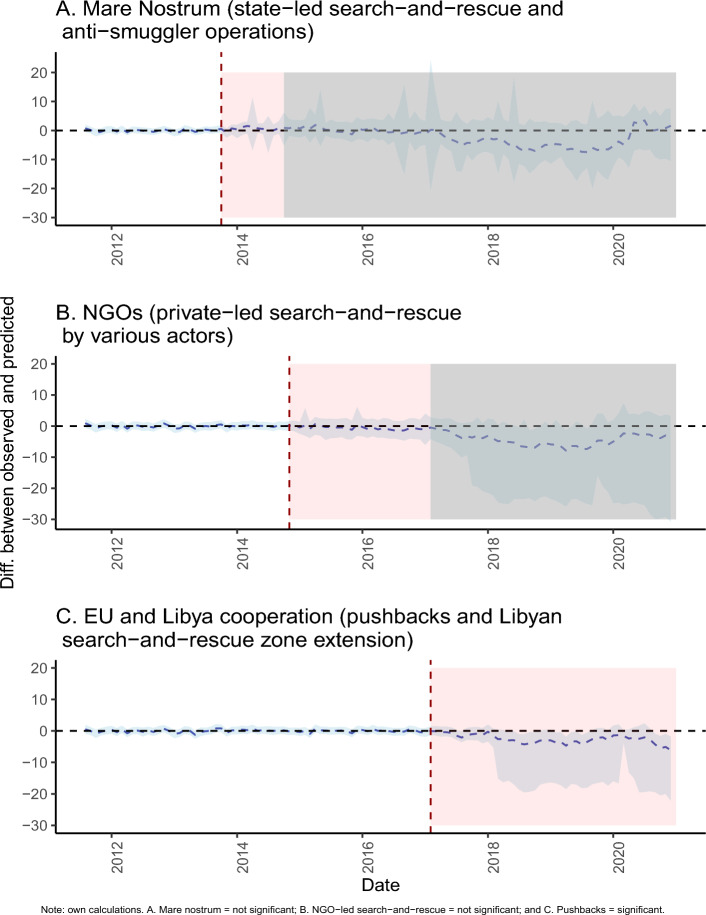
Pointwise effects (difference between observed and predicted) for our three intervention periods, 2011–2020.

Figure 2A shows that the observed trend after the start of *Mare Nostrum* remains within the confidence intervals of our predicted counterfactual. The only remarkable difference between the observed and the predicted series occurs around the Spring-Summer of 2014 when the observed trend is slightly *higher* than the predicted counterfactual, though the difference is not statistically significant—and which appears as a slight bump aroud that time in Fig. 3—where we show the difference between the observed and our counterfactual prediction. Moreover, we do not observe substantial changes in the seasonality of the predicted time-series with respect to the observed values, this is a remarkable feature of our model in light of important variation in seasonality over time in the original time series. Moreover, We emphasize here that *Mare Nostrum* was not just a search-and-rescue operation. One major component of the operation was the disruption of smugglers practices^34^, which had important impacts on the smuggling business in Libya. Besides rescuing migrants, *Mare Nostrum* targeted and criminalized smugglers and destroyed many of the wooden vessels used for transportation^35^.

A more straightforward test of the ‘pull factor' claim is shown in Panel B, also in Fig. 2, where the intervention period of private-led search-and-rescue is examined. Coordinated private-led search-and-rescue by NGOs materialized after the end of *Mare Nostrum*. In the corresponding post-intervention period, the observed and the predicted counterfactual time-series follow each other closely for nearly two years, and especially during the months where private-led search-and-rescue was the most intense (see Supplementary Materials S1). There is no discernible difference in the seasonality of our counterfactual series with respect to the observed one either. This empirical evidence directly contradicts the ‘pull factor' claim given that the difference between the prediction and observed series is far from statistically significant. In other words, based on our selection of *other* ‘push-and-pull factors', a similar number of crossing attempts was to be expected during the period where private-led search-and-rescue was the most active. In fact, for more distant periods after the start of private-led search-and-rescue, especially around mid-2017, our model predicts the opposite: that the number of crossing attempts should have been higher than the observed one, which would indicate that the period of private-led search-and-rescue reduced crossing attempts - though this difference is not statistically significant.

By contrast, as shown in Panel C, the surplus in the predicted counterfactual time-series shown in Panel A and B coincides, instead, with the start of the EU-Libya cooperation - captured in all counterfactual predictions, for the Mare Nostrum and NGOs-led search-and-rescue periods. For the EU-Libya cooperation intervention period, our prediction suggests a substantially higher number of crossing attempts than what was actually observed^28^. Given that the extension of the Libyan search-and-rescue area affected smugglers operations at the expense of a potential increase in the riskiness of the crossing^34,36^, the number of crossing attempts should have been much higher than observed. As shown in Panels A and B though, this higher number of crossing attempts could not have been a result of state- nor private-led search-and-rescue operations.

Synthesizing the previous findings, Fig. 3 shows the difference between the original and the predicted number of crossing attempts (in logs) - the pointwise effects. For the search-and-rescue periods (i.e., Fig. 3A,B), the pre- and post-intervention pointwise effects gravitate tightly around zero, as suggested by the previous discussion of no statistically significant differences. However, for the EU and Libya cooperation intervention period, the effect is slightly negative, which is suggestive of a reduction in the number of crossing attempts as a result of the coordinated pushbacks and the extension of the Libyan search-and-rescue zone. We note that this came at the expense of significant deterioration of the human rights situation of migrants in Libya^6,7,37^.

## Discussion

We have shown that the inflow of migrants along the CMR is not affected by the presence of search-and-rescue operations, as proposed by proponents of the ‘pull factor' claim. Although human mobility is indeed affected by various structural intervening-obstacles^19^, search-and-rescue activities, which may reduce the salience of the Mediterranean Sea as one important intervening-obstacle, cannot explain increases in the number of departures and arrivals coming from Libya, nor why people decide or are forced to move in the first place. Importantly, we did not find a substantial difference between the observed flow of migrants and our predicted counterfactual flow during the state-led and private-led search-and-rescue intervention periods even after various years from the start of the most salient search-and-rescue interventions. In other words, search-and-rescue does not seem to be a further *causal* driver of migration and, therefore, on average, does not incentivize more crossings. Although it may seem counter intuitive at first to some—especially because search-and-rescue periods do coincide with a relatively low mortality rate and a high number of arrivals, search-and-rescue operations do not affect an already existing flow, nor the stock of potential or prospective migrants willing to make the crossing. As hinted by previous research, search-and-rescue is a response to the higher flow, not a cause. Our results are in line with previous descriptive research on the role of search-and-rescue operations^9,23,24^, and, generally, with the study of migration drivers^29^—which suggest that irregular migration is far better explained by worsening economic conditions, environmental degradation, conflicts or violence, and political persecution, as partly captured by some of our predictive covariates.

In addition, our study highlights that the extension of the Libyan search-and-rescue area, in coordination with the EU, a cooperation which increased the leverage of the LCG in the area, did affect the number of arrivals to Europe, although many still attempt the crossing despite the increasing difficulty^6^. This result lends further credibility to our identification strategy and in particular the possibility to detect measurable effects with our approach. However, it is known that the reduction in the number of crossing attempts caused by the intervention of the LCG comes at the expense of well-documented human rights violations against prospective migrants, occurring in the process of pushback interventions and at detention centers along the Libyan coast^7,37^. Although externalization policies may serve the purpose of deterring or rerouting irregular migration, at least in the short run, such policies do not affect the structural drivers that influence a given flow^29^, and may force prospective migrants to take more dangerous routes.

The main results of this paper do contrast, however, with analyses based on micro-economic behavior models of migration—grounded in untested assumptions about unobservable characteristics of this market and each type of agent. Such studies find that search-and-rescue operations led to more crossing attempts and a higher risk of death^10,22^. However, one major drawback of the market-based interpretations of search-and-rescue effects—which ultimately rely on aggregate data and the use of questionable instrumental variables (see Table S7 in Supplementary Materials for a discussion of these)—is the inherent lack of an appropriate empirical counterpart with individual-level data corresponding to its underlying assumptions. For example, little is known about the relative safety of different types of boats^21^ and, importantly, we lack information about the decision margin of migrants in selecting boat type and the timing of departure, prices charged to migrants, types of markets where smugglers operate, costs of smugglers operations, etc.^34^. Therefore, serious questions about the micro-foundations of theoretical models, supposedly giving theoretical weight to the ‘pull factor' claim, remain. Generally, by relying on aggregate data (i.e., counts of crossings or deaths at a daily, weekly, or monthly time scale), one cannot possibly recover policy effects on individuals, which is the reason why we take a data-driven approach and interpret our findings as aggregate trends. Notwithstanding such evident lack of micro-level data, multiple studies make claims about how policies have affected prospective migrants and smugglers’ incentives, aspirations, and behaviors. Such claims are exemplary cases of the well-known ecological fallacy^38^, they disregard the potential for heterogeneous responses—resulting from existing differences among migrants^39^, and oversimplify the complex web of relationships between changes in maritime border policing, smugglers strategies, and migrants’ aspirations and capabilities^36^.

Employing various known ‘push-and-pull factors', we were able to build a credible counterfactual trend that tracks the observed flow of crossing attempts along the CMR with high accuracy, successfully predicting multiple features of the irregular flow of migrants in this route (e.g., trends, spikes, drops, seasonality). Other studies, similarly mixing traditional and non-traditional data and new technologies of data collection to better understand migration^40^, use a similar approach in migration-related and asylum-related management processes, for example, in the creation of warning systems^41,42^. However, predicting migration flows has been a difficult task for researchers^43^. Although other studies have attempted this^44,45^, we are the first to use predictive modeling to answer a specific causal inference question in migration research. Other predictive machine learning models could be compared to test for the best possible model, but this task is beyond the scope of this paper and such alternative predictive models should account for all the time series features of migration flows, and the high dimensionality of the predictive factors set. Our approach employs an existing, highly flexible model for time series forecasting, integrating predictive modeling, non-traditional digital data sources, and causal inference^46^. The ability of our models to predict various features of this time series behavior employing our selected covariates does show migration flow along the CMR is a function of various known ‘push-and-pull factors' operating in a dynamic, highly complex fashion at origin, residence, transit, and destination countries. Our predictions, nevertheless, are of the *ex post* type, meaning that we aim to predict a series of events which already took place. We do this with the goal of estimating causal parameters of interest, which makes our predictive model unsuited for predicting future flows or examining other causal inference questions.

The BSTS models we present here provide a fully Bayesian time-series estimate for the effects of interest; and perform a substantial dimensionality reduction through model averaging and covariate selection to re-create the most appropriate synthetic control to be used as a model for the different counterfactual scenarios. One advantage of this statistical modeling approach is that we do not need to make strong assumptions on individual-level, unobservable behavior, assumptions that oversimplify the complexity of migration movements for causal identification^22,47^. The most important assumption on which our results depend is that the control series are exogenous to the interventions of interest. In Supplementary Materials S1, we performed a series of tests to determine the extent to which this assumption might be violated. We find that our results are not sensitive to the exclusion of labor market indicators from the BSTS models - the only potentially relevant set of control series that could act as mediator in the causal process. Furthermore, we also find that there are only few spurious statistically significant correlations between our interventions and the control series that are implausible to be affected by changes in search-and-rescue.

Notwithstanding the advantages of our model, one shortcoming of our approach is that the post-intervention periods in Fig. 2 are also affected by later interventions, so that we cannot identify the delayed-effects in later time intervals when other interventions were in place. Our models predict well for a fixed and limited number of post-intervention months in the future (e.g., approximately two years or so), but the predictions—especially in Fig. 2A—become more unreliable as we move away from the start of the intervention of interest (i.e., larger variance and wider confidence intervals). Therefore, extrapolating the initial intervention to later periods, where another intervention was in place, is not warranted. Furthermore, data on arrivals to Europe through irregular border crossings, pushbacks, and deaths of migrants on transit suffers from underregister and underreporting, and therefore can only be considered as a lower-bound^1^. Although we have improved our measurement of crossing attempts by combining various data sources, the measure may still be lower than the actual number, which is unknown. There are not many studies addressing the particular issue of underreporting of deaths^24^, which, under this causal framework, may lead to biased inference. A recent study suggests that around 10% of deaths might have gone unrecorded by the various projects registering migrant deaths^48^. In terms of magnitude, however, the estimated number of deaths only constitutes around 4% of the total arrivals and pushbacks for the period of interest, meaning that underregister is unlikely to affect the behavior of the overall time series. Even though the absolute number of deaths is high, in relation to the magnitude of the number arrivals and pushbacks, deaths are a much smaller component of our crossing attempts measure. Therefore, this issue is unlikely to affect the main trends found in our study (e.g., a preliminary analysis of a correction for underreporting in deaths shows no deviations from our findings, these results are available upon request). Nonetheless, further research is needed on the subject of underreporting. Moreover, data on illegal border crossings and pushbacks to Libya and Tunisia might be affected by the number of officials patrolling the border and registering the numbers of migrants arriving in Europe or attempting to cross. It is plausible that there has been an increase in the number of officials over time, but unclear how they might affect the numbers reported. Our results constitute thus a conservative estimate of the intervention effects if our measure of crossing attempts is severely under-counted, and the underregister is not uniform over time.

A further limitation of our study is that we were unable to adjust for a potentially relevant feedback mechanism between search-and-rescue activities and smugglers operations (e.g., when a boat in distress calls the search-and-rescue boats in the area, this may further lead to other boats making distress calls after knowing the presence of a search-and-rescue boat in the area). However, such a feedback mechanism is not supported by existing evidence. While our coarse monthly data does not allow us to detect such patterns—which are likely to operate within a period of days or at most weeks—more detailed analyses of specific cases of NGOs involved in search-and-rescue, and employing geo-located and temporal data, suggest that there is no systematic connection between the rate at which smugglers send boats, the timing of distress calls, and the presence or absence of search-and-rescue boats in the area^49^. Finally, we are unable to determine, at the micro-level, whether an awareness of search-and-rescue operations could affect the decision making of migrants—in so far as it may affect their risk perception and mobility aspirations^50^. However, various organizational aspects of irregular migration—the recruitment, the choice of point of departure, time of departure, type of boat, number of people per boat, who gets to be when and in which boat, etc.– are entirely determined by smugglers, and not by migrants themselves^34^. Therefore, search-and-rescue operations are unlikely to drive the individual decision making processes of migrants, though they may influence smugglers’ decisions, something which is left for further research.

The images of mostly European humanitarian workers *pulling* out of the water mostly Sub-Saharan migrants about to drown has proven an irresistible metaphor for some. However, we must resist the use of such simplifying metaphors not only because they dehumanize migrants and contribute to the criminalization of search-and-rescue activities at sea^9^, but also because they are inadequate to describe the phenomenon of irregular migration in the CMR. Mechanical metaphors continue to play a prominent role in migration policy making and discussion, but they oversimplify the complexity of migration determinants and drivers^32^. We invite others to think about migration from a different perspective, and to unveil the coated ways in which certain rhetoric forces us to think about migration in relation to state bordering policies^51^.

## Methods

### Data

The main dependent variable in our study is the monthly number of crossing attempts through the CMR $Y_t$ in the period 2010–2020, which roughly captures the magnitude of irregular migration movements (i.e., crossing attempts). As a measure of arrivals, we added the number of illegal border crossings reported by FRONTEX, the number of forcibly returned migrants as reported by the Tunisian and Libyan Coast Guards, as well as the number of documented deaths. For this last component, we compile the most comprehensive data on mortality that is available, employing UNITED for Intercultural Action - campaign office ‘Fortress Europe No More Deaths’^52^, the Migrant Files^53^, and the Missing Migrants Project^54^. Further clarifications on sources used can be found in Supplementary Materials S1. Each of the components of crossing attempts—arrivals, pushbacks, and deaths—may be measured with error, the number of deaths $D_t$ is likely to be systematically affected by underreporting^1^.

In order to accurately predict the migration flow across the CMR and to distinguish the effects of different intervention periods on this flow, we consider various ‘push-and-pull factors' that may explain this flow^29^. We combine multiple sources of information, as shown in Table S1 in Supplementary Materials, which provides an overview of the variables used in our study, including definition, time period, source, and operationalization. Given that the flow of migrants corresponds to what the literature refers to as mixed migration (i.e., migrants who may escape persecution in their home country and/or who seek better economic prospects), in our predictive model, we include many factors known to be associated with the inflow of migrants along this route (i.e., with the restriction that all control time-series should be observed for the whole period of interest, and with a time granularity of minimum monthly data). We based many of the chosen indicators on multiple factors affecting the flow from Africa to Europe^55^, such as conflicts and economics crises. The complexity of irregular migration from West and North Africa across the Mediterranean to Europe requires a consideration of the multiple linkages between migrants’ socioeconomic and demographic characteristics and the experienced social circumstances in their countries of origin. Social networks and diasporas abroad are a further important predictor of migration flows^56^. However, in our case, given our focus is not on specific country-to-country flows, diaspora effects are less relevant, which is why we did not include them in our model.

Although we are unable to capture changes on complex processes of development and social transformations that may lead to changes in migration flow^57^, our adjustment time-series set captures various economic, violence or conflict, and environmental time-series presumably affecting this mixed-migration flow^58^. We employ various economic time-series to build our predictive counterfactual: the African and MENA region currency exchange rates with the EURO (European Central Bank Statistical Data Warehouse), which may capture reductions in remittances at a finer time scale; the economic series of international commodity prices compiled by the International Monetary Fund (IMF) for more than 30 commodities (including energy sources, such as coal, crude oil, gas, propane, various agricultural products and fertilizers, and multiple base and rare metals); and the unemployment rate in the EU and specific European countries (Eurostat). The time-series on three weather variables captured in Malta and Italy consists of recorded temperature, precipitation, and number of storms. As further 'push factors', we included additional economic time-series. Given the lack of updated and historical data on unemployment in Africa, we used as indicators of labor market behavior Google Trend Searches for the terms “job”, “work”, and “employment” in Arabic for North African countries due to lack of comparable official data on unemployment rates. We employ time-series on conflicts and violence from the ACLED data for African countries^59^. Given that the Syrian conflict was also particularly important in the inflow, but given lack of data in the same time frame and scale we are interested in, we also employed Google Trends Searches on Syrian conflict to measure the intensity of the conflict as reported in online search activity. Moreover, we use an indicator for environmental disasters, including both natural and technological disasters, occurred in African countries as a further 'push factor' from a well-known and comprehensive database^60^. Finally, given that many other factors affecting the inflow were not observed, we include the Airport flows from African and MENA region countries to Europe (https://www.sabre.com/). With this measure we intend to capture movements related to other causes not captured by our covariates. Air traffic flows have been used to estimate transnational human mobility worldwide^61^. By incorporating the number of passengers through airflow—which are unrelated to the flow through the sea—we can adjust for further unobserved factors affecting irregular migration flows towards Europe to some extent. As stated above, an important assumption of the BSTS model is that covariate ‘push-and-pull factors' or predictive time-series should be unaffected by the different interventions, which in our case we consider is highly plausible (e.g., see robustness checks in Supplementary Materials S1). Finally, the information on the presence of search-and-rescue NGOs’ boats was based on a careful reading and combination of multiple sources, and is documented in Supplementary Materials S1.

We use monthly series for the period of 2011–2020. When data was available at a smaller than monthly time scale, such as days or weeks, we aggregated the time-series to the month by computing the average or simply the sum of observed values. We computed various lags of the adjustment time-series $\mathbf {X_t}$, of up to six months (i.e., $t-j$ for $j=1,2,3,4,5,6$), and these lagged variables were also used as adjustment time-series. All our variables were merged using the month and year dates as identifying key variables. Our data contains no serious missing information problems, but when a few single points of data in a given series were missing, we employed single imputation using a Kalman filter to predict the single missing data point^62^—this was the case for two dates on our weather variables. For the prediction exercise, we employ a log transformation of the number of crossing attempts to deal with changes in the variance over the observation window^63^, which contains periods of both increasing and decreasing variance. This transformation of our dependent variable has a stabilizing effect on our target series, therefore improving the prediction ability of our models. Such a transformation has been shown to substantially reduce the mean squared error (MSE) in forecasting models, when it stabilizes the time-series variance^63^.

### A counterfactual approach for interventional effects on migration flow time-series

Data available for the study of the ‘pull factor' claim are discrete time-series counts, so we restrict our analysis to the aggregate-level, and refrain from making claims about the unknown micro-motives of migrants and smugglers, e.g., ref.^22^. If the ‘pull factor' claim holds, however, we would expect to see changes in the aggregate flow as expected by previous studies in the direction of a higher number of crossing attempts in periods where systematic search-and-rescue efforts were active. Although looking at changes in the levels of flows make intuitive sense, past research has not considered other forms in which there can be an upsurge in crossing attempts in the absence of search-and-rescue operations—which is the truly counterfactual causal inference question at play.

First, we employ a time-series decomposition method to describe the general trend observed in the time-series of crossing attempts, as well as the potential for a seasonal component in it^33^; and subsequently we perform a test for structural changes, based on the simultaneous estimation of multiple breakpoints^64^, to describe whether changes in the flow correspond to important changes in the politics of search-and-rescue (see Supplementary Materials S1 for these analyses). Second, we employ a Bayesian structural time-series (BSTS) statistical model to build a synthetic counterfactual time-series of crossing attempts (following each intervention) that accounts for potential changes in trends, seasonality, repeating and nested patterns, as well as spikes or drops in the target time-series^65^. The aim is then to compare the observed number of crossing attempts to the synthetic or predicted counterfactual time-series.

This BSTS model constitutes a generalization of the difference-in-differences and synthetic control causal inference designs in the time-series context^30,65^. The BSTS model is based on a diffusion-regression state-space model which consists of an observation and a state equations. The observation equation is given by $Y_t = {Z_t}^T \alpha _t + \varepsilon _t$, where $\alpha _t$ is a *d*-dimensional state vector, $Z_t$ is a *d*-dimensional output vector, $\varepsilon _t \sim N(0, \sigma _t)$ is an error term and *d* is the number of factors or components believed to be associated with the target series $Y_t$. This observation equation links the observed time-series $Y_t$ to the latent state space variable $Z_t$, which determines the behavior of the series in the post-intervention period. The state equation, in turn, is given by $\alpha _{t+1} = T_t \alpha _t + R_t\eta _t$ where $T_t$ is a $d \times d$ transition matrix, $R_q$ is a $d \times q$ control matrix, and $\eta _t \sim N(0, Q_t)$ is another error term. The multiplication of the error structure $R_t\eta _t$ with the control matrix is suited for models where there are more covariates than time-points, for the case when the matrix of state components would be less than full rank, as in our case. Models incorporating a seasonality component are an example a state-space time-series model and the estimation of the model parameters is based on a stochastic algorithm for posterior inference based on Markov chain Monte Carlo (MCMC)^30^.

These models are more adequate than other predictive time-series approaches in various ways^66^, and have been shown to better capture the temporal evolution of the impact of interventions^30^. Multiple sources of variance—including local linear trends, a seasonality component, and time-invariant as well as potentially time-varying contemporaneous covariates—can be flexibly accommodated by further setting $Z_t = \beta _t \mathbf {X_t}$ and $\alpha _t=1$, which takes into consideration the effect of covariates or predictive factors through a linear regression written in state-space form. In this way, we can additionally account for variance components shared between these control or adjustment time-series $\mathbf {X_t}$ and our target series $Y_t$ (see Table S1 Supplementary Materials for an overview of these factors). This is particularly important in successfully predicting the flow of crossing attempts along the CMR in the post-intervention period—which in our case is the out-of-sample predictions, given that, as discussed in the background section, crossing attempts depend on various other ‘push-and-pull factors', which may reflect in the series with a delay of months or even years. Importantly, these control time-series, which act as predictive factors, should not be affected by the changes in the politics of search-and-rescue in order for them to build a reliable counterfactual unaffected by policy changes, which we believe is the case. A comparison of alternative ML models for time series is beyond the scope of this study, but remains an important component of predictive modeling for causal inference in migration research. Other projects should evaluate which ML model is better capable of predicting an even more trustworthy counterfactual on the basis of its fit in the pre-intervention period (see Supplementary Materials S1). In Supplementary Materials S1, we perform model validation employing pre-intervention data, exploring how the predictive performance of our BSTS models changes when adding or deleting some of the state-space specifications, and diagnostic plots for distributions of residuals which are suggestive of a good fit of the final model.

Finally, from an initial set of predictive factors deemed relevant for building a predictive counterfactual time-series - for theoretical or empirical reasons, not all covariates ought to necessarily be included in the matrix $\mathbf {X_t}$. In fact, including them all may lead to noisy and thus unreliable predictions. This means that, within one predictive model, a form of model selection can be performed on the control time-series covariates to improve the model’s fit. This is done by further including a spike-and-slabs prior into the state-space model specification, which adds penalties on the parameters of each covariate^30^ based on how well they actually predict the target series in the pre-intervention period. The spike-and-slabs prior puts a certain amount of posterior probability at zero for regression coefficients that are not predictive. The algorithm uses an MCMC for fitting and we employ 10,000 iterations. Overall, the BSTS model employs the pre-intervention period covariate information and their association to the log of crossing attempts to create a predictive model, and then uses the fitted model to predict the future crossing attempts in the post-intervention period. The method is fundamentally a way of predicting the counterfactual, and therefore unobserved, time-series of crossing attempts had there been no changes in the politics of search-and-rescue during the three intervention periods under consideration. These counterfactual time-series are built on a set of candidate predictor or adjustment/control time-series that together build a single synthetic control^67^. The differences between the observed and the counterfactual series—predicted by the BSTS model—are considered as the treatment effect.

## Supplementary Information

Supplementary Information.

## Data Availability

We document the various data sources used in Table S1 in Supplementary Materials. Though most data sources are publicly available—with the exception of the Sabre data on air traffic, we are unable to upload our data set to a repository due to data-usage requirements and proprietary restrictions. The data that support the findings of this study are available from various sources documented in Table S1 in Supplementary Materials but restrictions apply to the availability of these data, which were used under license for the current study, and so are not publicly available. Data are however available from the authors upon reasonable request and with permission of the various third party owners of the data. The code to construct the data set and perform the various statistical analyses is available at https://github.com/xlejx-rodsxn/sar_migration.
