# Supplementary material for: Search-and-rescue in the Central Mediterranean Route does not induce migration: Predictive modeling to answer causal queries in migration research
Source: Sci Rep. 2023 Aug 3;13:11014. doi: 10.1038/s41598-023-38119-4 (PMC10400626; doi:10.1038/s41598-023-38119-4)
Supplement: Supplementary file 1 — Supplementary Information. [file 41598_2023_38119_MOESM1_ESM.pdf]

Search-and-rescue in the Central Mediterranean Route does not induce migration: Predictive modeling to answer causal queries in migration research (SUPPLEMENTARY MATERIALS)

Data

Table S1 presents the variables used to construct our target time series of attempted crossings, as well as the covariates used in our prediction model, together with their sources and operationalization.

Table S1: Description of variables used in the study and sources

Category	Variable	Period	Scale	Unit	Definition	Source
Arrivals, Crossings and Deaths	Asylum applications to EU	2008-2021	Monthly	Number of persons	First-time applicants for asylum by applicant's age and citizenship and gender	Eurostat (https://ec.europa.eu/eurostat/web/migration-asylum/asylum/database)
	Illegal border crossings (FRONTEX)	2009-2021	Monthly	Number of attempted crossings	Detections of illegal border-crossing along the main migratory routes into the EU - Black Sea Route; Central Mediterranean Route ; Circular Route from Albania to Greece; Eastern Borders Route; Eastern Mediterranean Route; Other; Western African Route; Western Balkan Route; and the Western Mediterranean Route.	Frontex Risk Analysis Network (FRAN) and Joint Operations Reporting Aapplication (JORA) data (https://frontex.europa.eu/we-know/migratory-map/)
	Arrivals to Italy and Malta	2015-2021	Daily, Monthly	Arrivals	Data on arrivals by sea to Italy and Malta, by date and place of disembarkment. Nationality of disembarked migrants and/or country of departure is reported. This information partial and only to be used as an indication.	IOM's Displacement Tracking Matrix Flow Monitoring Europe - Central Mediterranean route
	Airport flows from African and MENA region countries to Europe	2011-2021	Monthly	Number of passengers	Historical air traffic data containing the number of passengers from origin countries to destination countries in a selection of countries from Asia, Africa, and Europe, processed and provided by SABRE.	Sabre Travel Data (https://www.sabre.com/)

Table S1: Description of variables used in the study and sources *(continued)*

Category	Variable	Period	Scale	Unit	Definition	Source
	"Pushbacks"	2016-2021	Monthly	Number of people	Interceptions by Libyan Coast Guard Interceptions by the Libyan and Tunisian Coast Guards. Reported negative values around the Spring of 2019 were recoded to positive (-2971 on 2019-06-01 and -2398 2019-05-01)	IOM's Missing Migrants Project' (https://missingmigrants.iom.int/downloads)
	Missing and Death IOM-MMP	2014-2021	Daily	Number of deaths	The number of deaths of migrants who have died or gone missing while travelling towards an international destination.	IOM's Missing Migrants Project' (https://missingmigrants.iom.int/downloads)
	The Migrant Files	2000-2016 (June)	Daily	Number of deaths	Compilation of various data sources. It uses UNITED's data base, Fortress Europe, and Puls. It also collects real-time from publicly available sources. Duplicates registered deaths were removed one by one	The Migrant Files (https://www.themigrantsfiles.com/)
	Deaths (UNITED List of Death)	1993-2021	Daily	Death count	Number refugee deaths collected from various network organisations in 48 countries, local experts, journalists, and migration researchers. We restricted the deaths to those recorded in a set of coordinates corresponding to the Central Mediterranean Route or that were classified as happening in any of the countries in this route. We did not exclude deaths.	UNITED for Intercultural Action's List of Refugee Deaths (https://unitedagainstrefugeedeaths.eu/about-the-campaign/about-the-united-list-of-deaths/)

Table S1: Description of variables used in the study and sources *(continued)*

Category	Variable	Period	Scale	Unit	Definition	Source
Push-and-pull factors	African and MENA region currencies' exchange rates with EURO	2010-2020	Daily	Euros per currency unit	Averaged monthly exchange rates of various Sub-Saharan African currencies and MENA region	European Central Bank Statistical Data Warehouse extracted with the PriceR package in R (https://cran.r-project.org/web/packages/priceR/priceR.pdf)
	Job Search Indicators in Africa (Google Trends)	2008-2020	Daily	Google Index	Given the absence of high granularity labor market data in North Africa, we use Google Trends to capture employment fluctuations by looking at trends over time for the terms job, work and employment in Arabic.	Google Trends, employing the gtrendsR package in R (https://cran.r-project.org/web/packages/gtrendsR/gtrendsR.pdf)
	Conflict indicators: battles, explosions, violence, protests, riots in Africa	2008-2020	Daily	Number of events	Data on the locations, dates, actors, fatalities, and types of all reported political violence and protest events in African countries. We employ number of battles, explosions or remote violence, protests and social unrest, military strategic developments, and violence against civilians.	ACLED (https://acleddata.com/#/dashboard)

Table S1: Description of variables used in the study and sources *(continued)*

Category	Variable	Period	Scale	Unit	Definition	Source
	Intensity of Syrian conflict (Google Trends)	2008-2020	Daily	Google Index	In contrast to African countries, ACLED did not cover the Syrian situation to the same extent. To counter this, we made use again of Google Trends for the "Syrian war", which captured the attention given to the conflict, and serves as a measure of the intensity of the conflict.	Google Trends, employing the gtrendsR (https://cran.r-project.org/web/packages/gtrendsR/gtrendsR.pdf)
	Commodity prices	2000-2020	Monthly	Prices	Monthly data on various primary commodity prices from four broad categories of energy, agriculture, fertilizers, and metals with base year 2016.	IMF (https://www.imf.org/-/media/Files/Research/CommodityPrices/TechnicalDoc.ashx)
	Environmental disasters	1998-2020	Daily	Events	Counts of natural and technological disasters with a geographical and temporal dimension. It provides information at the country level.	EM-DAT, The International Disaster Database (https://public.emdat.be/)
	Weather variables	2010-2020	Monthly	Degrees, cubit meters, and day counts	Average temperature, average precipitation and the number of storms in Mediterranean weather stations in Italy and Malta	European Climate Assessment & Dataset project (https://www.ecad.eu/)
	Unemployment rate in EU and specific countries	2008-2022	Monthly	Rate	Unemployment rates in the EU and EA, seasonally adjusted.	Eurostat (https://ec.europa.eu/eurostat/web/lfs/data/database)

Table S1: Description of variables used in the study and sources *(continued)*

Category	Variable	Period	Scale	Unit	Definition	Source
Other	European Union Operations	2009-2021	Daily	Binary	Binary indicators taking the value of one when operations were active and zero otherwise.	Own elaboration based on official sources.
	NGOs search-and-rescue mission dates	2009-2021	Daily	Binary	Own elaboration from multiple sources.	See Table 2 in the next section.

Note: Own elaboration.

Dates of search-and-rescue NGO-led operations and EU-led operations

Our information on the dates of search-and-rescue NGO missions taking place - including the days when a rescue mission started and when vessels were being held in a harbor or ending a mission - is collected from multiple sources. The first source of information was the official websites of the NGOs, where sometimes this information was made available. However, given that only few of them provided this information, we made used of published news prints and research articles containing the exact days. This helped to get the schedules of the different search-and-rescue missions. Although at times these strategies did not provide exact dates, they gave us with at least a rough estimate of the time frame. This estimate was further used as a reference period to further search in other sources. Many of the NGOs engaged in search-and-rescue missions make use of social media platforms, like Twitter, to raise awareness of the events occurring in the Mediterranean ocean, inform their supporters of their activities, or just collect funds for future rescue attempts. Given that based on the estimated time frame, we knew they were deployed in the central Mediterranean Sea, we made use of the information in social media platforms as a second direct source of information. If it was not possible to find dates through any official sources the research had to be widened to include secondary more indirect sources like interviews with NGO officials or reports in newspapers. Together these three different sources provided the exact dates of start, end and (involuntary) breaks in the several search-and-rescue missions in the Mediterranean Ocean. Binary indicators taking the value of one when the NGO was active in the Central Mediterranean and zero otherwise.

Table S2: Dates of NGO-led search-and-rescue operations

NGO	reason	Date	Source
SeaWatch	start of mission	"20.06.2015"	https://sea-watch.org/ueber-uns/5-jahre/
	break	"02.07.2018" - "20.10.2018"	https://sea-watch.org/sea-watch-3-verlaesst-malta
	pause	"31.01.2019" - "22.02.2019"	https://sea-watch.org/sea-watch-3-auf-dem-weg-in-planmaessigen-werftaufenthalt-in-frankreich/
	pause	"18.05.2019" - "01.06.2019"	https://doi.org/10.24989/0014-2492-2019-34-155
	pause	"29.06.2019" - "30.12.2019"	https://sea-watch.org/sea-watch-startet-nach-sechsmonatiger-blockade-in-rettungsmission/
	pause	"28.02.2020" - "06.06.2020"	https://sea-watch.org/sea-watch-3-rettet-ueber-90-fluechtende-vor-der-libyschen-kueste/
	pause	"17.06.2020" - "20.09.2020"	https://sea-watch.org/sea-watch-3-startet-rettungseinsatz/
	end of mission	"26.03.2021"	https://sea-watch.org/rettungsschiff-sea-watch-3-erneut-festgesetzt/
ProActiva Open Arms	start of mission	"05.07.2016"	https://twitter.com/openarms_fund/status/750313484529860608
Salvamento Maritimo Humanitario	start of mission	"01.10.2018"	https://www.diariodenavarra.es/noticias/navarra/2018/09/27/dya-navarra-crea-proyecto-atencion-personas-refugiadas-613052-300.html
	start of pause	"18.01.2019"	https://www.lasexta.com/noticias/sociedad/el-aita-mari-segundo-barco-humanitario-al-que-niegan-permiso-para-salir-hacia-libia-video_201901185c423ea70cf2ab9ab5a448b0.html#
	end of pause	"17.04.2019"	https://www.infomigrants.net/en/post/16402/spanish-ngos-to-deliver-aid-supplies-to-greek-islands
	pause	"08.05.2020" - "08.12.2020"	https://projekte.sueddeutsche.de/artikel/politik/seenotrettung-im-mittelmeer-2020-die-bilanz-e670960/
Sea Eye	start of mission	"19.04.2016"	https://twitter.com/seaeyeorg/status/865094856309452800
	start of pause	"21.06.2018"	https://sea-eye.org/en/alan-kurdi-anchors-off-sardinia/

Table S2: Dates of NGO-led search-and-rescue operations *(continued)*

NGO	reason	Date	Source
	end of pause	"21.12.2018"	https://m.tagesspiegel.de/vor-der-kueste-libyens-die-rueckkehr-der-zivilen-seenotretter/23799480.html
	start of pause	"05.05.2020"	https://sea-eye.org/en/2020/05/
	end of pause	"11.09.2020"	https://sea-eye.org/en/alan-kurdi-returns-to-sea/
	start of pause	"25.09.2020"	https://sea-eye.org/en/alan-kurdi-anchors-off-sardinia/
	end of pause	"08.05.2021"	https://sea-eye.org/en/on-the-way-to-save-lives/
Mare Liberum	start of mission	"26.08.2018"	https://www.facebook.com/MareLiberumOfficial/photos/a.198832504178994/285431368852440/
	pause	"23.04.2019" - "13.05.2019"	https://mare-liberum.org/en/mare-liberum-siegt-vor-gericht/
	pause	"19.08.2020" - "02.10.2020"	https://twitter.com/teammareliberum/status/1311988014348894208
Mission Lifeline	start of mission	"20.09.2017"	https://mission-lifeline.de/mission-1-3/
	pause	"08.11.2018" - "18.11.2017"	https://mission-lifeline.de/mission-5/
	pause	"25.11.2017" - "13.06.2018"	https://mission-lifeline.de/mission-6/
	start of pause	"17.10.2018"	https://mission-lifeline.de/mission-7/
	end of pause	"26.08.2019"	https://mission-lifeline.de/erster-einsatz-fur-eleonore/
	end of mission	"02.09.2019"	https://mission-lifeline.de/eleonore-legt-nach-8-tagen-in-sizilien-an
Mediterranea	start of mission	"03.10.2019"	https://mediterranearescue.org/en/news-en/the-mare-jonio-is-welcomed-by-mediterranea-activists-as-it-arrives-in-venice-for-maintenance-work/
	pause	"19.03.2019" - "27.03.2019"	https://www.ilpost.it/2019/03/27/mare-jonio-dissequestro-migranti
	start of pause	"10.05.2019"	https://orf.at/stories/3121695/
	end of pause	"02.07.2019"	https://mediterranearescue.org/en/news-en/we-left-the-port-mediterranea-is-back-at-sea/

Table S2: Dates of NGO-led search-and-rescue operations *(continued)*

NGO	reason	Date	Source
	start of pause	"06.07.2019"	https://www.zeit.de/gesellschaft/zeitgeschehen/2019-07/mediterranea-alex-rettungsschiff-notstand-lampedusa-hafen-italien
	end of pause	"22.08.2019"	https://orf.at/stories/3121695/
	pause	"02.09.2019" - "04.02.2020"	https://mediterranearescue.org/en/news-en/mare-jonio-confiscation-and-fine-of-300-000e-absurd-and-inhuman-application-of-security-decree-bis/
	pause	"19.03.2020" - "09.06-2020"	https://projekte.sueddeutsche.de/artikel/politik/seenotrettung-im-mittelmeer-2020-die-bilanz-e670960/
	end of mission	"25.09.2020"	https://mediterranearescue.org/en/news-en/rescue-forbidden-mare-jonio/
M.V. Louise Michel	start of mission	"22.08.2020"	https://www.united4rescue.com/download/U4R_Fotobuch_An_Bord_Die_erste_Mission_der_Sea-Watch_4_powered_by_United4Rescue.pdf
	end of mission	"22.10.2020"	https://twitter.com/mvlouisemichel
Refugee Rescue	start of mission	"January 2016"	https://www.refugeerescue.org/our-history
	end of mission	"14.08.2020"	https://www.refugeerescue.org/latest-news/statement-suspension-of-operations
Migrant Offshore Aid Station	start of mission	"26.08.2014"	https://www.facebook.com/migrantoffshoreaidstation/posts/today-marks-5-years-since-our-first-sar-mission-in-the-mediterranean-since-then-/1089709054551109/
	end of mission	"06.09.2017"	https://www.moas.eu/de/moas-stoppt-einsatz-im-mittelmeer-und-startet-mission-fuer-rohingya-fluechtlinge-suedostasien/
Jugend Rettet	start of mission	"24.07.2016"	https://jugendrettet.org/de/about#our_story
	end of mission	"01.08.2017"	https://jugendrettet.org/de/about#our_story
SOS Méditerranée & Médecins Sans Frontières	start of mission	"07.03.2016"	https://sosmediterranee.com/chronology-not-letting-anyone-drown-6-years-of-sos-mediterranee/
	start of pause	"20.11.2018"	https://www.msf.org/sinister-attacks-italian-authorities-lifesaving-search-and-rescue-mediterranean
	end of pause	"21.07.2019"	https://www.msf.org/msf-resumes-search-and-rescue-amid-deteriorating-conditions-libya-mediterranean-migration

Table S2: Dates of NGO-led search-and-rescue operations *(continued)*

NGO	reason	Date	Source
	start of pause	"20.03.2020"	https://sosmediterranee.com/to-our-supporters-ocean-viking-docked-in-marseille-teams-remain-fully-mobilized/
	pause	"22.06.2020"	and https://sosmediterranee.com/mission-report-starting-on-june-22nd-2020-sos-mediterranee-resumes-life-saving-mission-at-sea/
	pause	"22.07.2020" - "11.01.2021"	https://sosmediterranee.com/press/the-ocean-viking-is-headed-for-the-central-mediterranean-after-half-a-year-of-imposed-blockade/
Médecins Sans Frontières	start of mission	"09.05.2015"	https://www.bourbonoffshore.com/en/offshore/bourbon-argos-mediterranean-rescue-operations
	start of pause	"15.01.2016"	https://www.bourbonoffshore.com/en/offshore/catching-bourbon-argos
	end of pause	"05.05.2016"	https://www.bourbonoffshore.com/en/offshore/bourbon-argos-mediterranean-rescue-operations-resume
	start of pause	"20.11.2016"	https://twitter.com/IsmanSara/status/800285762637930496
	end of pause	"20.03.2017"	https://www.aerzte-ohne-grenzen.de/unsere-arbeit/aktuelles/mittelmeer-zwei-rettungsschiffe-geburt-baby
	end of mission	"05.10.2017"	https://www.msf.org/mediterranean-msf-ends-mission-search-and-rescue-boat-prudence
Save the Children	start of mission	"08.09.2016"	https://www.savethechildren.org/us/about-us/media-and-news/2016-press-releases/save-the-children-s-search-and-rescue-ship-sets-sail-from-sicily
	start of pause	"24.11.2016"	https://twitter.com/MP_SaveChildren/status/801720054291267584
	end of pause	"19.04.2017"	https://www.savethechildren.org/us/about-us/media-and-news/2017-press-releases/save-the-children-brings-300-refugees-and-migrants-to-safety-as-
	end of mission	"23.10.2017"	https://palermo.repubblica.it/cronaca/2017/10/23/news/migranti_perquisita_la_nave_di_save_the_children_ormeggiata_a_catania [illegible] 179105860/

Note: Own elaboration.

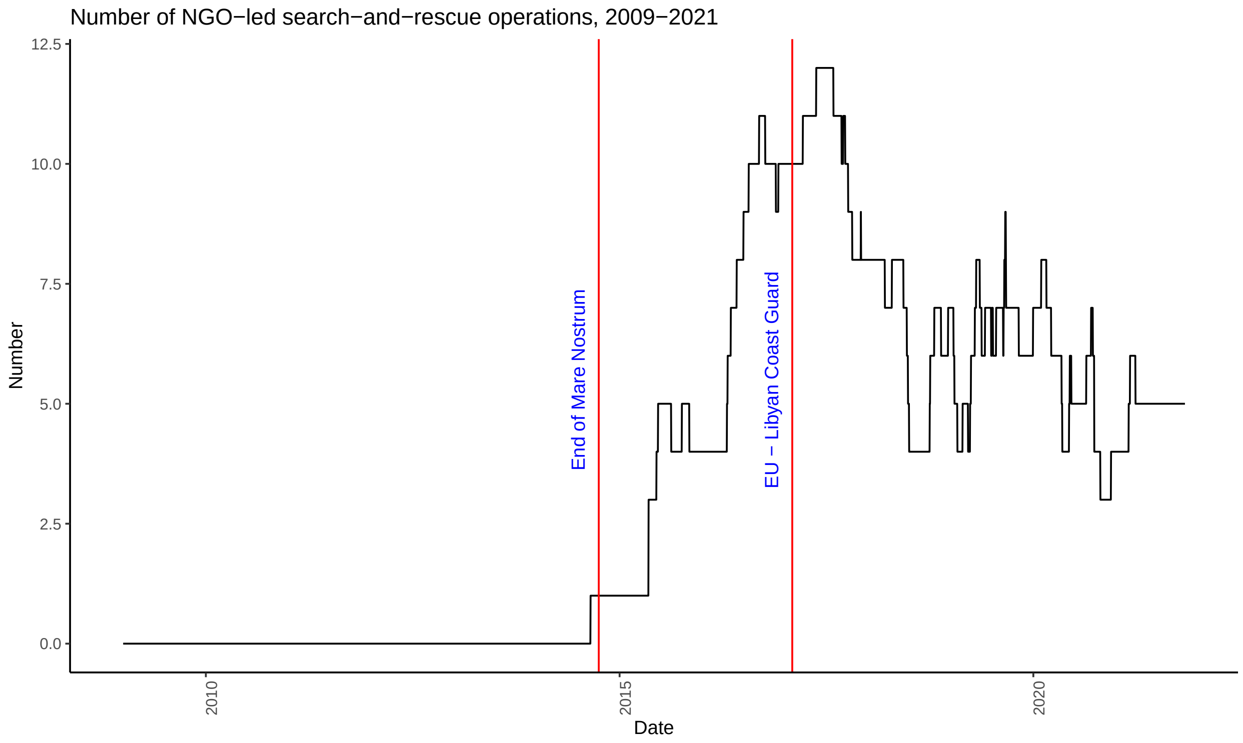

Figure S1: Number of NGO-led search-and-rescue operations, 2009-2021

Time series decomposition of attempted crossings

Our decomposition of the time series shows important seasonal and trend components. This in turn suggest that a causal inference model for the effect of interventions on attempted crossings (log) should account for these components in order not to bias the estimates.

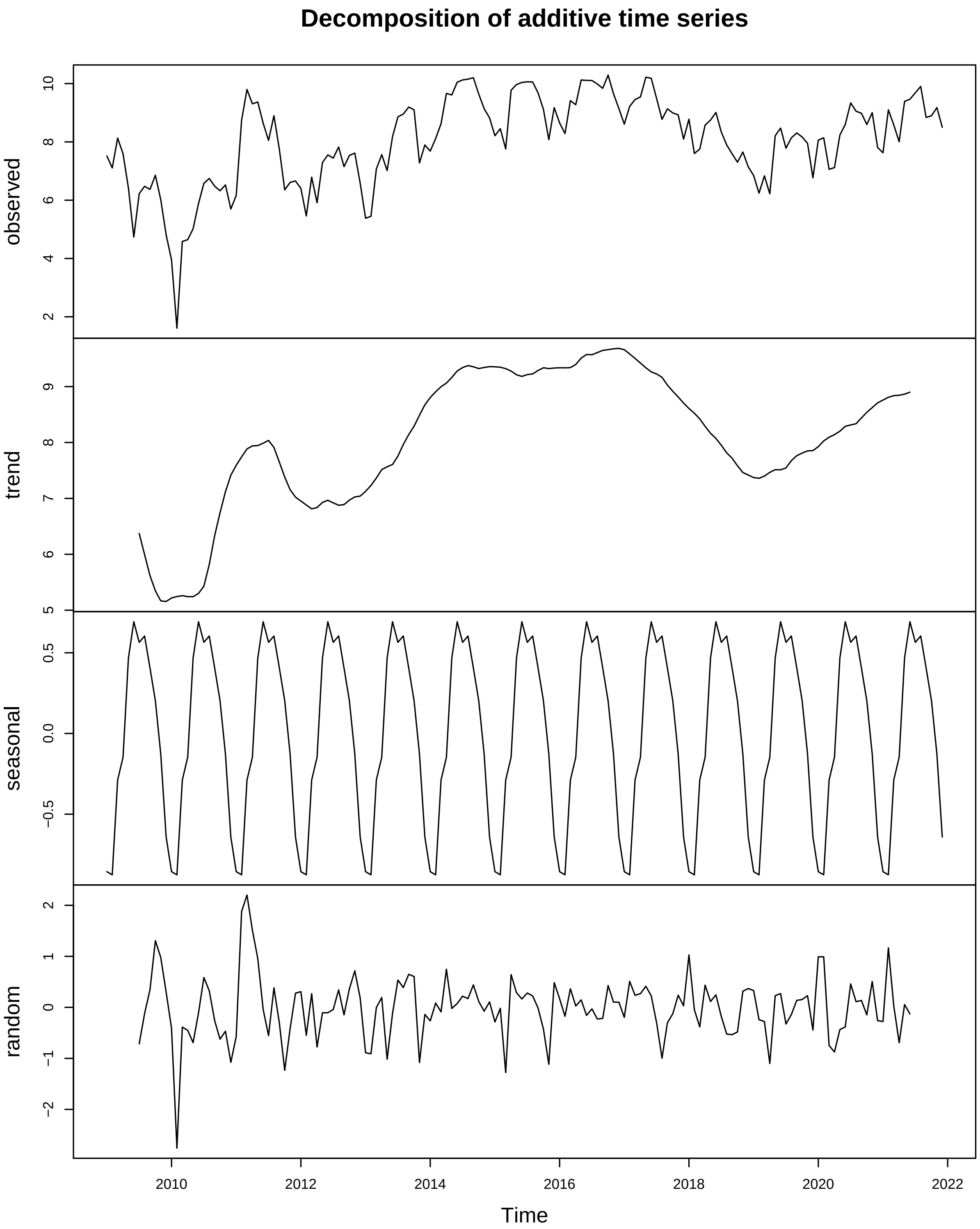

Figure S2: Decomposition of log of attempted crossings across the CMR

Deads and missing count

One important factor affecting our measure of attempted crossings is the underregister of deaths. The true number of deaths in the CMR is an unknown quantity that may affect our estimates[1]. In Figure S3, we show the evolution of the ratio of the sum of known deaths and missing migrants over the sum of arrivals and pushbacks along the CMR for the period of study. This figure suggest that deaths are a relatively small component of our measure of attempted crossings, particularly so during our intervention periods. In a recent study[2], underregister was found to be of a relatively small magnitude: less than 10% of deaths are estimate to have gone unrecorded. It is, however, unclear how this underregister changes over time, which is crucial to evaluate how it might affect our estimates.

It might well be that underregister is higher during the intervention periods under study, but the data in Figure S6 suggest that before search-and-rescue activities the ratio of deaths and missing was far higher than during search-and-rescue activities, when the ratio seems to be in fact much lower and around 0.1. Equally so after the start of the cooperation between the Libyan Coast Guard and the European Union. There is reason to believe that search-and-rescue activities in fact could reduce underregister of deaths given that more efforts are made to search for fatalities, thus improving reporting in a more systematic fashion. Hence, mostly the pre-intervention period would be affected by underregister. We believe this is unlikely to affect our estimates given that our BSTS models would be able to correctly capture the pre-intervention dynamics with the true unobserved number of deaths.

Similarly to the main study on the effects of the three intervention periods – The Mare Nostrum period, the private-led search-and-rescue, and the period of coordinated pushbacks made possible by EU-cooperation with the Libyan Coast Guard, we perform additional analyses of the time series of number of estimated deaths[3]. The results of these analyses show important changes in the count of dead and missing migrants during the period of interest. Although we attempted to duplicate the prediction exercise for this count, this time series is much more difficult to predict with our covariates, probably due to shipwrecks being a function of many unobserved factors and a combination of random events (e.g., changes in weather, type of boat, number of people per boat, experience of drivers, and many other circumstances). Preliminary results of

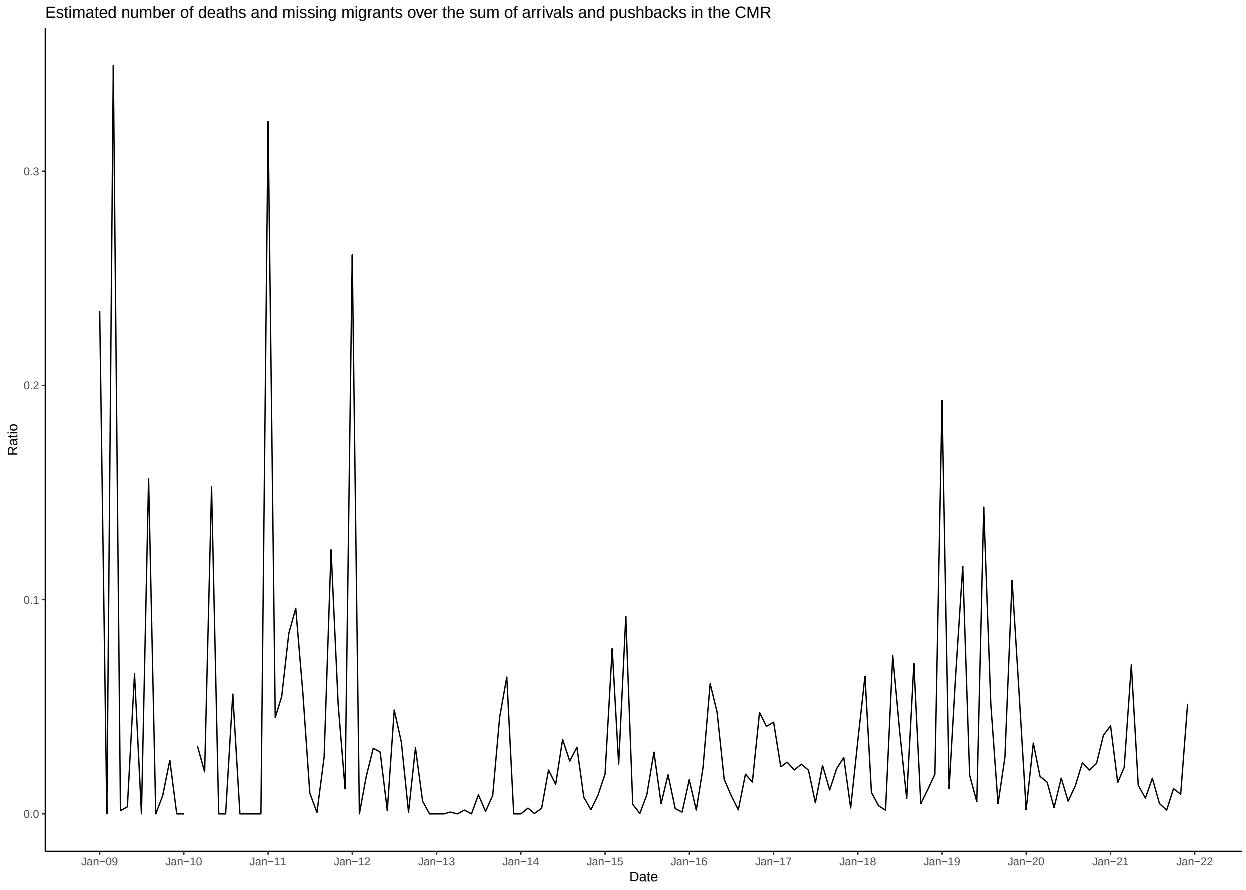

Figure S3: Ratio of estimated deaths and missing migrants over the sum of arrivals and pushbacks in the CMR

these models are available upon request. Therefore, predictive models for estimated deaths are not equally well suited to infer the effect of the three interventions periods on the number of deaths along the CMR.

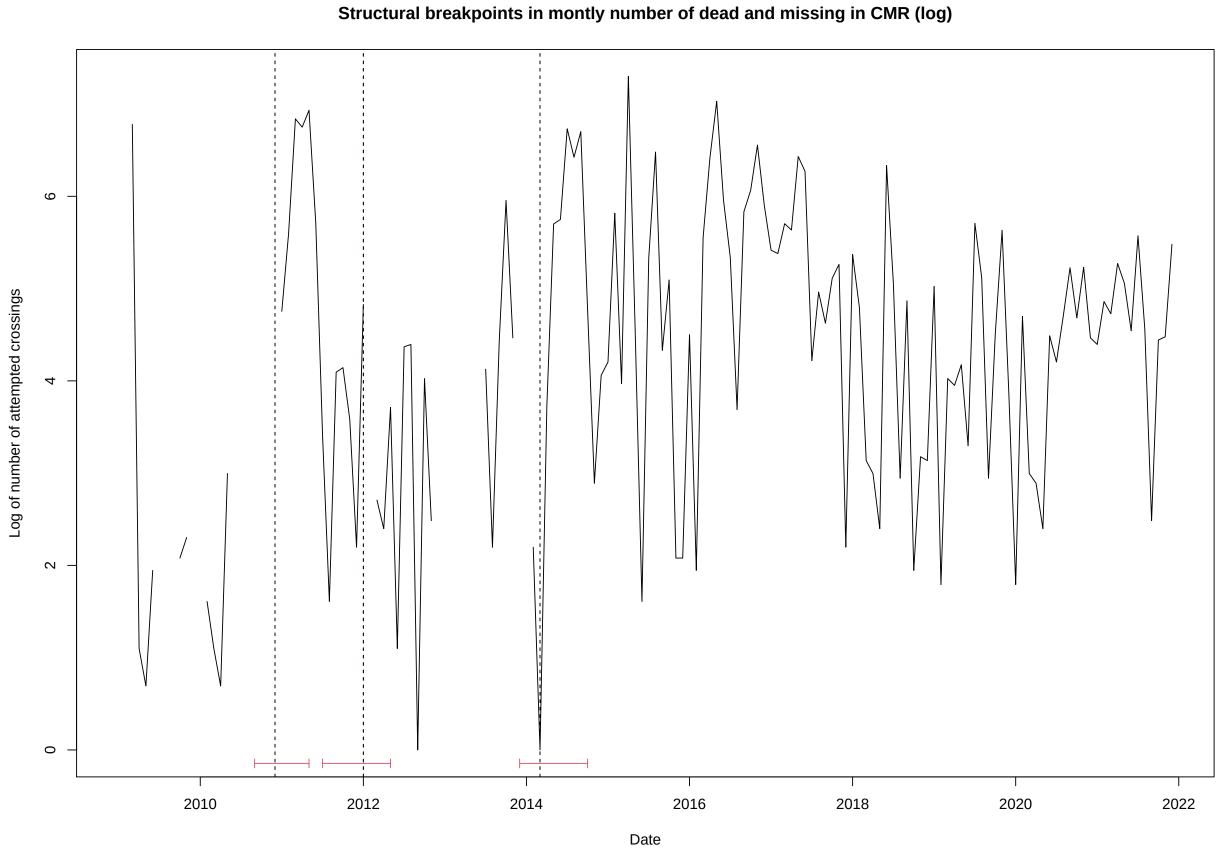

Figure S4: Structural breakpoints in montly number of dead and missing along the CMR (log)

Structural breakpoints in time series of attempted crossings

We performed a test for structural changes in the time-series of attempted crossings, based on the simultaneous estimation of multiple breakpoints[3]. This analysis reveals four distinct breakpoints or changes in the behavior of the time-series of attempted crossings and show important changes in the behavior of the target time series. The breakpoints, shown as dotted dark red vertical lines, roughly follow important changes in the politics of search-and-rescue in CMR, but they do not coincide with our intervention periods. The first break-point occurs in 2011, when a considerable change in the trend, in comparison to the previous two years, is observed, and which might be related to the start of the Syrian civil war. The second break-point occurs a few months after the start of the *Mare Nostrum* period, whereas the third break-point seems to capture the start of cooperation between the EU and the LCG and the extension of the search-and-rescue zone of the LCG. These breakpoints, though merely descriptive, are suggestive of policy change effects. Finally, the fourth structural break-point, which takes place in mid-2020, instead, might reflect the effects of the COVID-19 pandemic crisis on the inflow along the CMR.

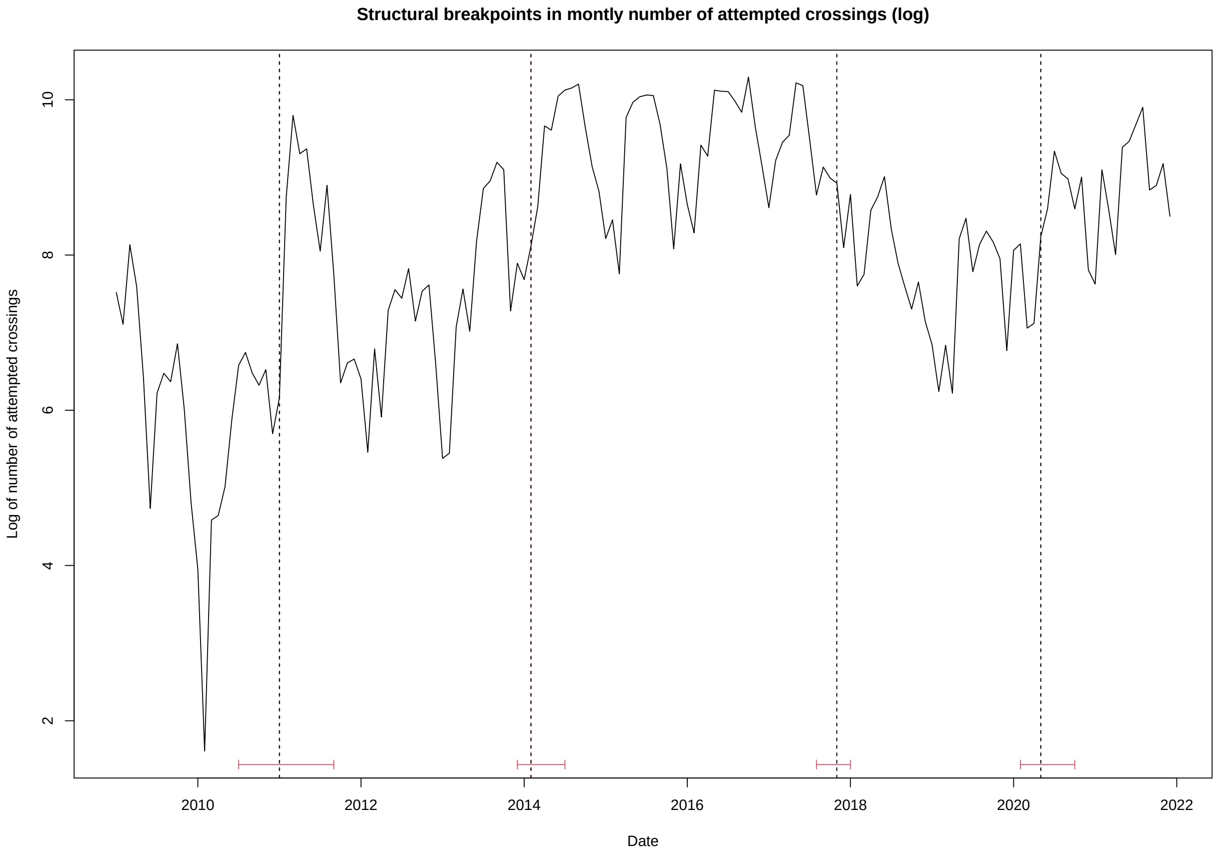

Figure S5: Structural breakpoints in montly number of attempted crossings (log)

Model validation and comparison with other machine learning models

Our causal identification strategy is based on a combination of the difference-in-differences and synthetic counterfactual frameworks[4,5], which, as argued in the main paper, is the most suited model for the type of question we try to answer given the data and intervention settings. In our case, where we have pre and post intervention periods with different regimes of search-and-rescue and pushbacks being active/inactive, we want to be able to establish a fair comparison while attending to basic elements of aggregate, discrete count time series data used for this (i.e., cycles or changes in variability, trends, seasonality, and a randomness).

The results of our BSTS models depend to an important extent on which state specification is used[5], and also, though to a lesser extent, on the selection of priors for many parameters. Model validation is therefore important for these state specifications. In particular, we perform model validation to evaluate the performance of these main components of the state specification in our BSTS models:

- The *local level* given by the equation $\alpha_{t+1} = \alpha_t + \epsilon_t$, with $\epsilon_t \sim N(0, \sigma)$; and where cross-validation is applied on a set of parameters for σ.
- The *local linear trend* component which assumes mean and slope of the trend follow a random walk, with equations for the mean given by $\mu_{t+1} = \mu_t + \delta_t + \epsilon_t$, with $\epsilon_t \sim N(0, \sigma_\mu)$, and for the slope $\delta_{t+1} = \delta_t + \eta_t$, with $\eta_t \sim N(0, \sigma_\delta)$.
- The *semi-local linear trend*, though equally assuming the same random walk process for the mean, assumes for the slope a stationary AR1 process centered on a value D, as expressed in $\delta_{t+1} = D + \phi(\delta_t - D) + \eta_t$ with $\eta_t \sim N(0, \sigma_\delta)$, which is particularly useful when making projections far into the future.

In hindsight, adding the seasonality component to the model made the fitting worse given that the seasonality, although present in the data, is not stationary, meaning that it changes over the period, and which correspond to an important feature of the target series to be predicted by our models. Therefore, we excluded this component from the final model.

We perform cross-validation using 5 folds and evaluate the prediction employing a different horizon length

depending on the availability of pre-intervention data (three months for the Mare Nostrum and six months for the private-led search-and-rescue and the extension of the Libyan search-and-rescue area). Given that we have three intervention periods and hence three different BSTS models, we perform model validation for each of these models employing only the pre-intervention data in each case. We evaluate the performance of the different parameter configurations employing three metrics: root mean squared error (RSME), the mean absolute prediction error (MAPE), and the mean absolute scaled error (MASE).

Table S3: Model comparison of validation metrics RMSE, MAPE, and MASE

Intervention	Models	RMSE	MAPE	MASE
Mare Nostrum	Local Linear Trend (LLT)	0.72	9.33	0.99
	Semi Local Linear Trend (SLLT)	0.75	9.34	1.03
	Local Level (LL)	0.75	9.59	1.03
	LLT + AR	0.80	10.33	1.10
	SLLT + AR	0.84	10.63	1.17
	LL + AR	0.90	11.12	1.20
	LL + SLT + AR	0.92	11.82	1.31
	Default: LL	1.08	13.28	1.48
NGOs	Local Level (LL)	1.12	13.14	1.68
	LL + AR	1.14	13.15	1.74
	Local Linear Trend (LLT)	1.41	16.98	2.13
	Semi Local Linear Trend (SLLT)	1.36	16.65	2.13
	Default: LL	1.41	14.94	2.14
	LLT + AR	1.40	17.13	2.17
	SLLT + AR	1.42	17.13	2.21
	LL + SLT + AR	1.53	18.34	2.38
EU and Libya cooperation	LL + AR	0.90	8.10	1.38
	Local Level (LL)	1.07	9.69	1.63
	SLLT + AR	1.13	10.10	1.71
	LL + SLT + AR	1.12	9.98	1.71
	LLT + AR	1.15	10.43	1.74
	Semi Local Linear Trend (SLLT)	1.16	10.39	1.76

Table S3: Model comparison of validation metrics RMSE, MAPE, and MASE *(continued)*

Intervention	Models	RMSE	MAPE	MASE
	Local Linear Trend (LLT)	1.34	12.23	2.05
	Default: LL	1.57	14.20	2.48

Note: Own elaboration.

The comparison of cumulative absolute error shown in Figure S6 reveals that the default model beats the cross-validated measures for the components of the state space model we focused on. This behavior is probably due to the short horizon on which we can evaluate the performance of the model. Our predictions for a causal effect extend over years and therefore the fit of the model should be evaluated on a much longer horizon. This, however, is not possible due to the limited availability of data.

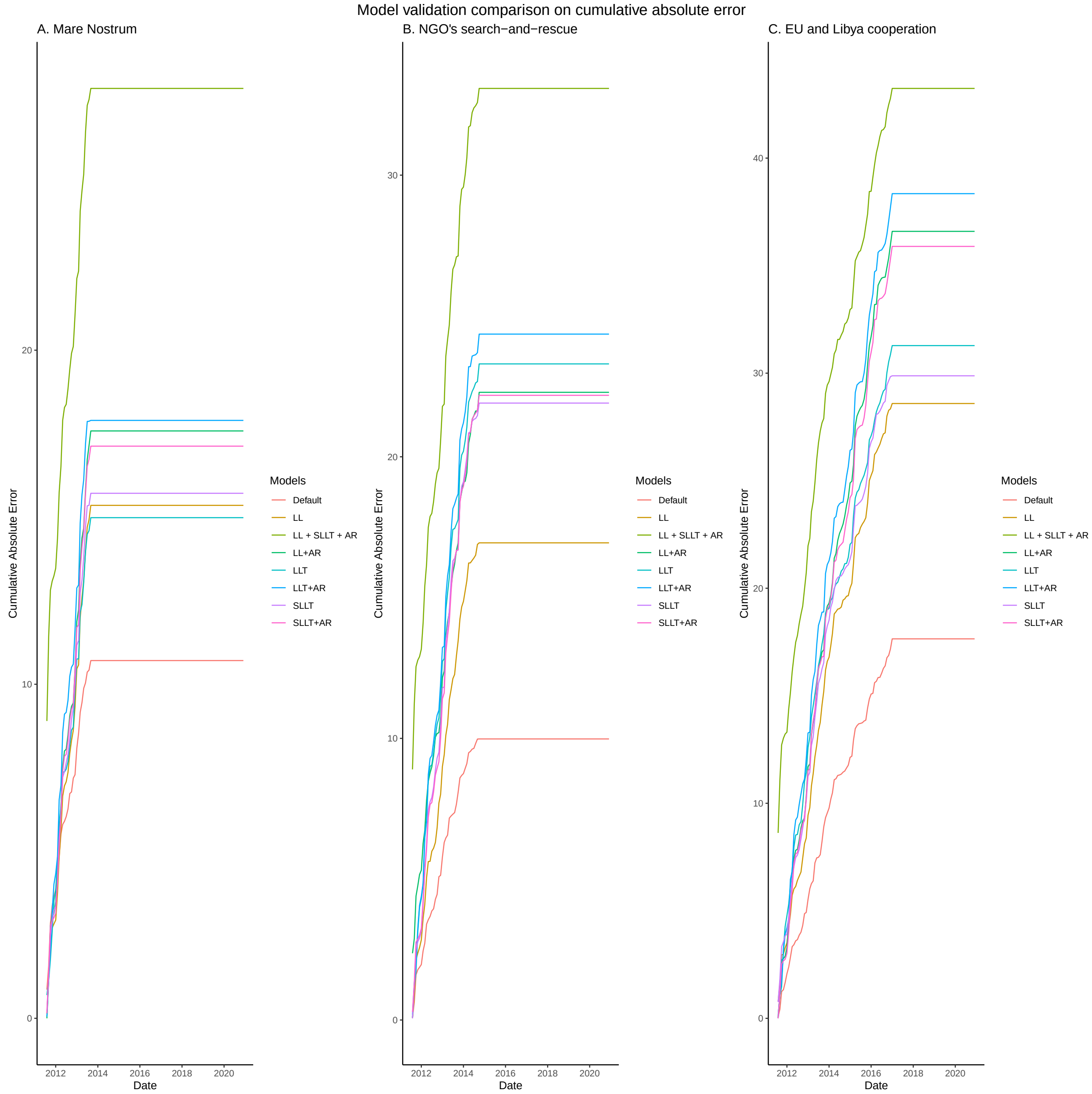

Figure S6: Model validation comparison on cumulative absolute error

Estimates of the different models, however, do not differ much as shown in Figure S7. The most notorious

difference between these estimates are the standard errors. Some models provide much wider CIs, but the effect is of a similar magnitude across models.

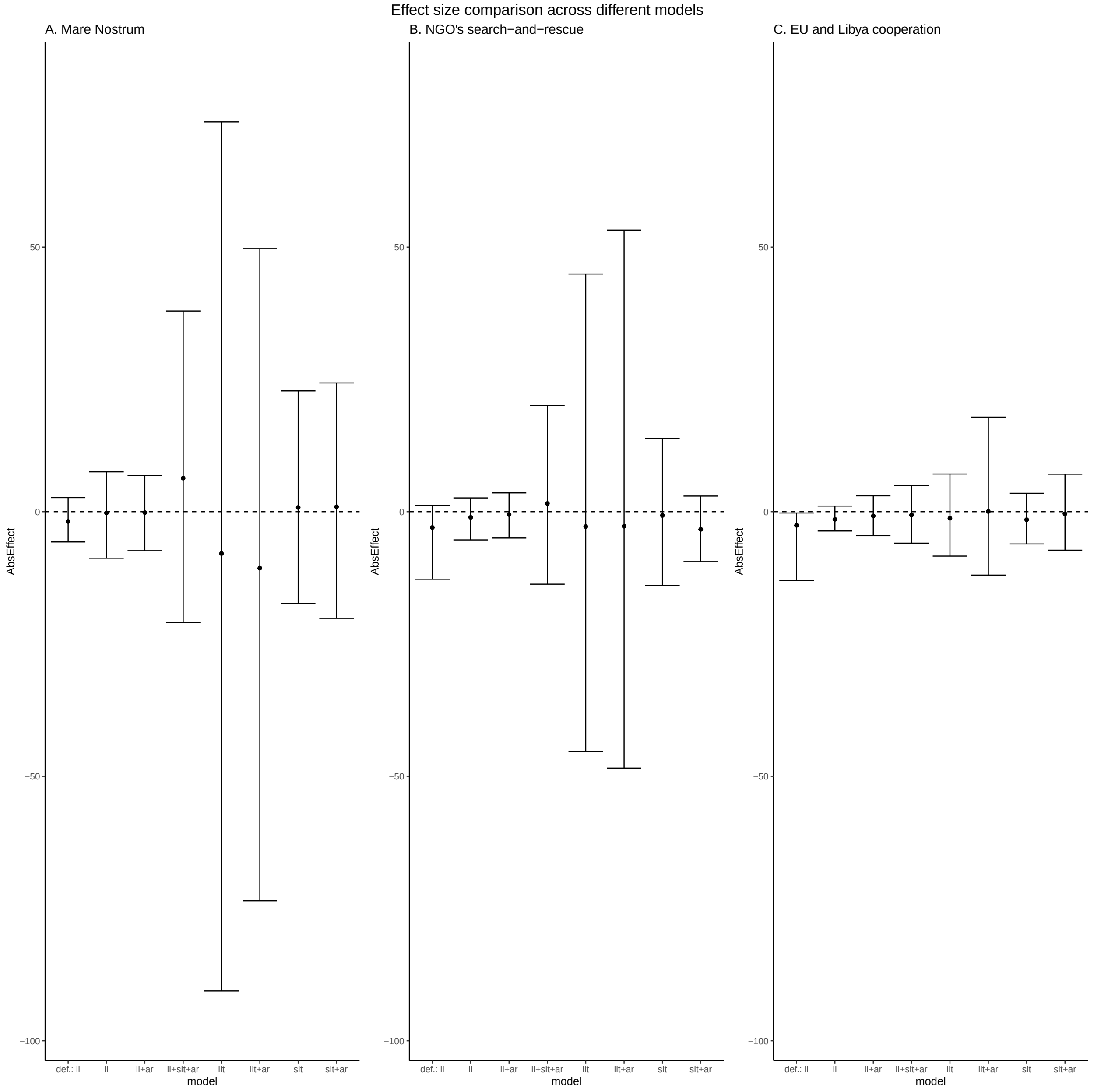

Figure S7: Effect size comparison across different models

The final models we present in the main paper have a relatively good fit as seen in the analysis of residuals presented in Figures S8 and S9. The autocorrelation plot in Figure S8 shows the characteristic drop towards zero.

Although the tails of the error distribution do not follow precisely on the straight line - probably due to

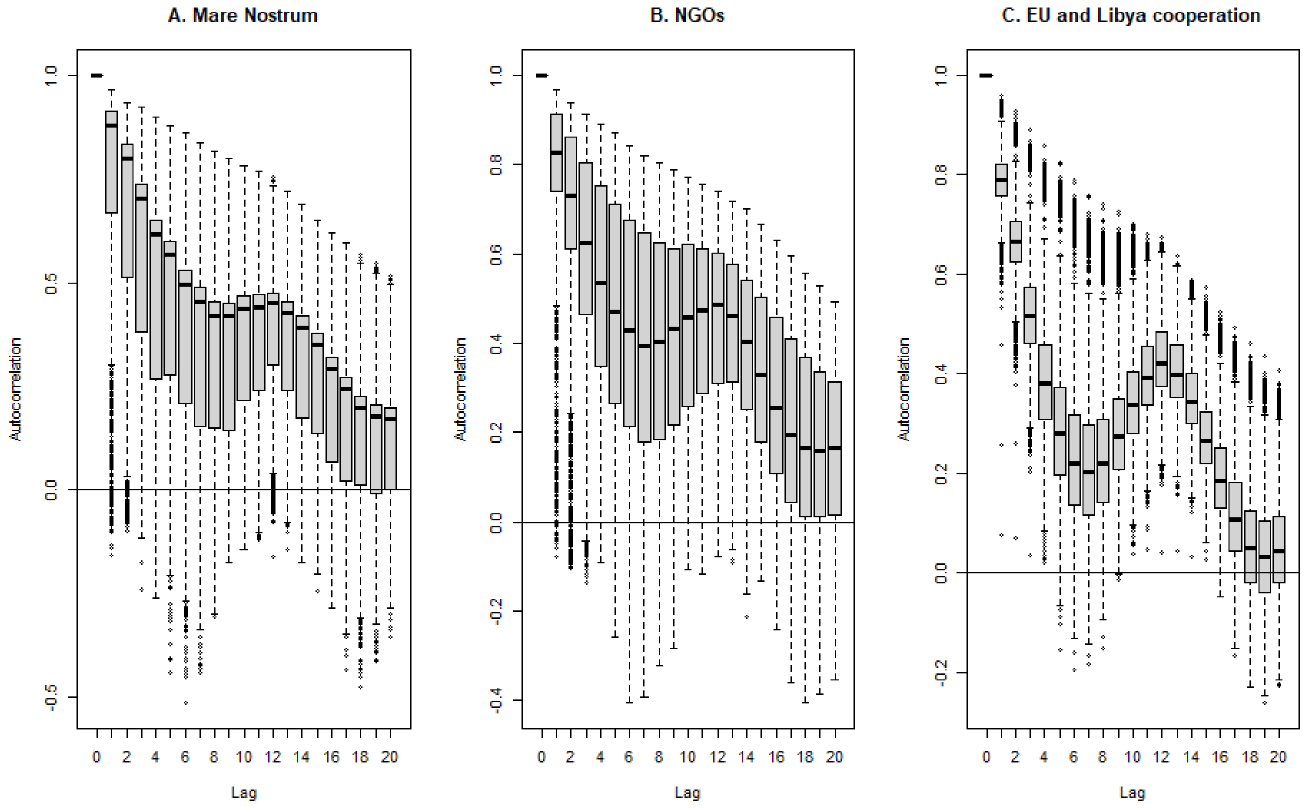

Figure S8: Autocovariance/autocorrelation function for the predicted series for the three intervention periods

variable omission, our models show a good fit in general and not a strong deviation from the assumed normal distribution for the residuals.

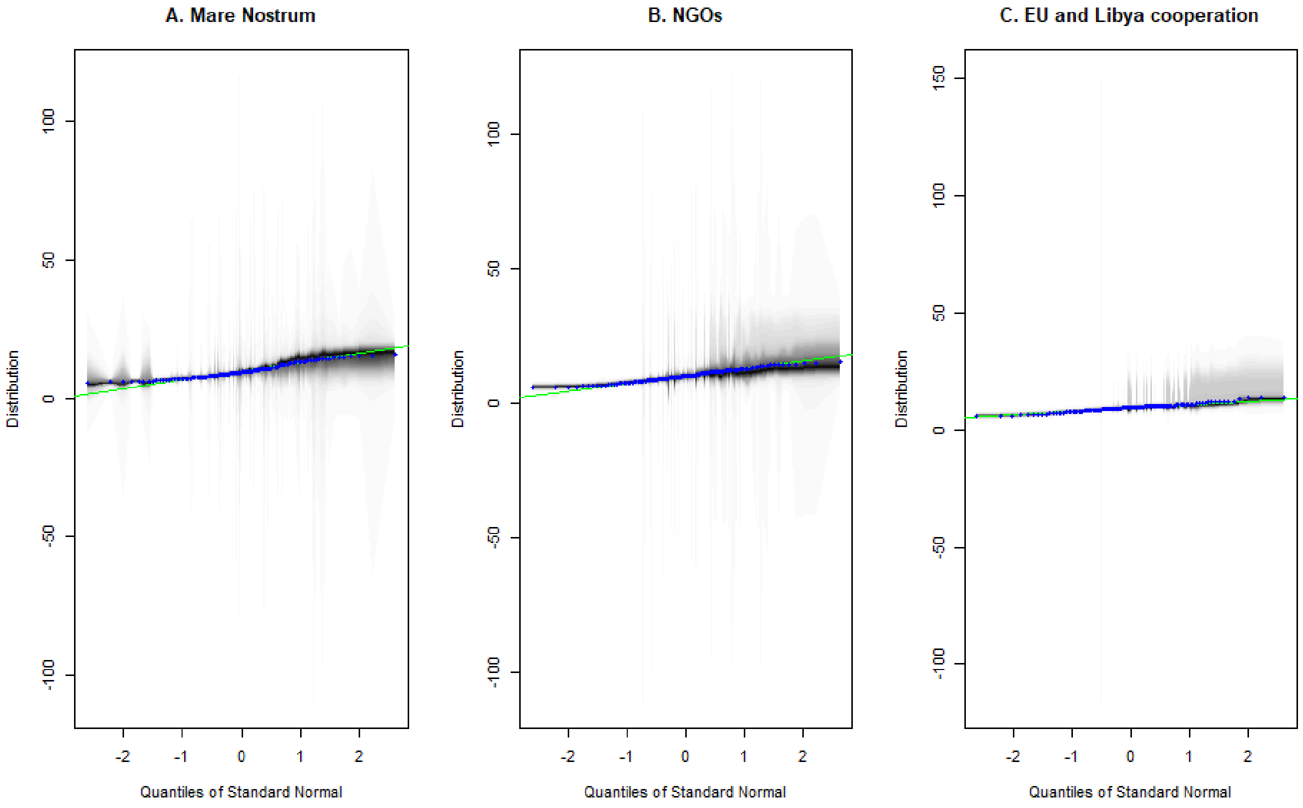

Figure S9: Quantile-quantile plot for the distribution of mean of Monte Carlo draws against the quantiles of the standard normal distribution

Selected covariates in BSTS final models

By means of spike-and-slab prior, for our three predictive models we have a subset of covariates for each intervention period which were deemed predictive. These are shown in Figure S3. The color of the bars representing the direction of the effect. For illustration, we present covariates with an inclusion probability of 10% or more, but other covariates with a lower inclusion probability and hence a lower weight were part of the model.

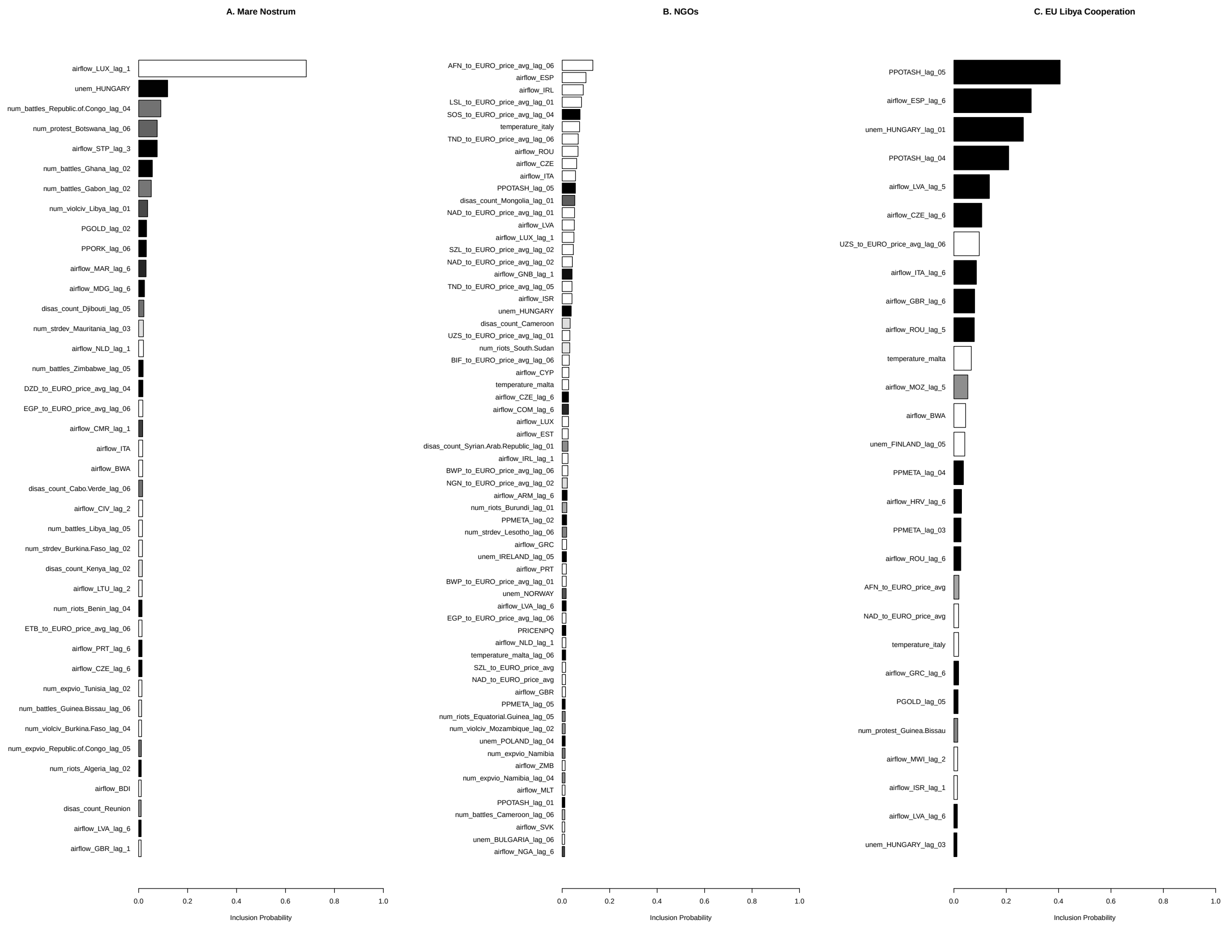

Figure S10: Selected covariates in each BSTS model for the three intervention periods

Mediation analysis

One of the most important assumptions of our approach is that the control series used in the BSTS model are exogenous with respect to the interventions of interest. If these control series were affected by any of our interventions, our identification strategy would be compromised, as we would be overcontrolling for potentially relevant elements of the causal process connecting changes in search-and-rescue politics and number of attempted crossings. In this section, we pay close attention to this issue by highlighting the potential for the labor market indicators (e.g., unemployment rates in Europe and job searches in North African countries) to be affected by the hypothetical "pull-factor" effect of search and rescue operations or the extension of the Libyan search-and-rescue area. We do this only for these variables because we believe these are the only ones we can plausibly see as potentially affected by the interventions in a feedback way (i.e., if search-and-rescue does constitute a "pull factor," more migrants will leave their country of origin or destination, reducing the labor supply in those labor markets, and more migrants will arrive to Europe, increasing the labor supply in corresponding local European labor markets). However, we do not believe the number of arrivals is large enough to have such disruptive effects on European or North African labor markets. In addition, there are physical and bureaucratic labor market access constraints for migrants entering Europe and seeking asylum.

In order to rule out this hypothetical mechanism, we exclude the labor market indicators from the set of control series out of which the BSTS model can select and re-run our models. The results of these models, shown in Figure S11, show very similar effects, as those including these series with only small variations in the predicted counterfactual, which makes intuitive sense given that each variable in our model has a minor contribution to the generation of the synthetic counterfactual.

Second, and to rule out potential mediators, we perform a series of bivariate Granger causality tests, which are a series of Wald tests comparing an unrestricted model – where the control series is explained by lags of up to sixth order of the control series and the intervention of interest – to a restricted model in which the control series is only explained by its lags. Given that we make multiple comparisons, we adjust the p-values employing the Bonferroni correction by dividing the significance levels by the number of tests being carried out - because of tests for reverse causality, this number is further multiplied by two. Results of

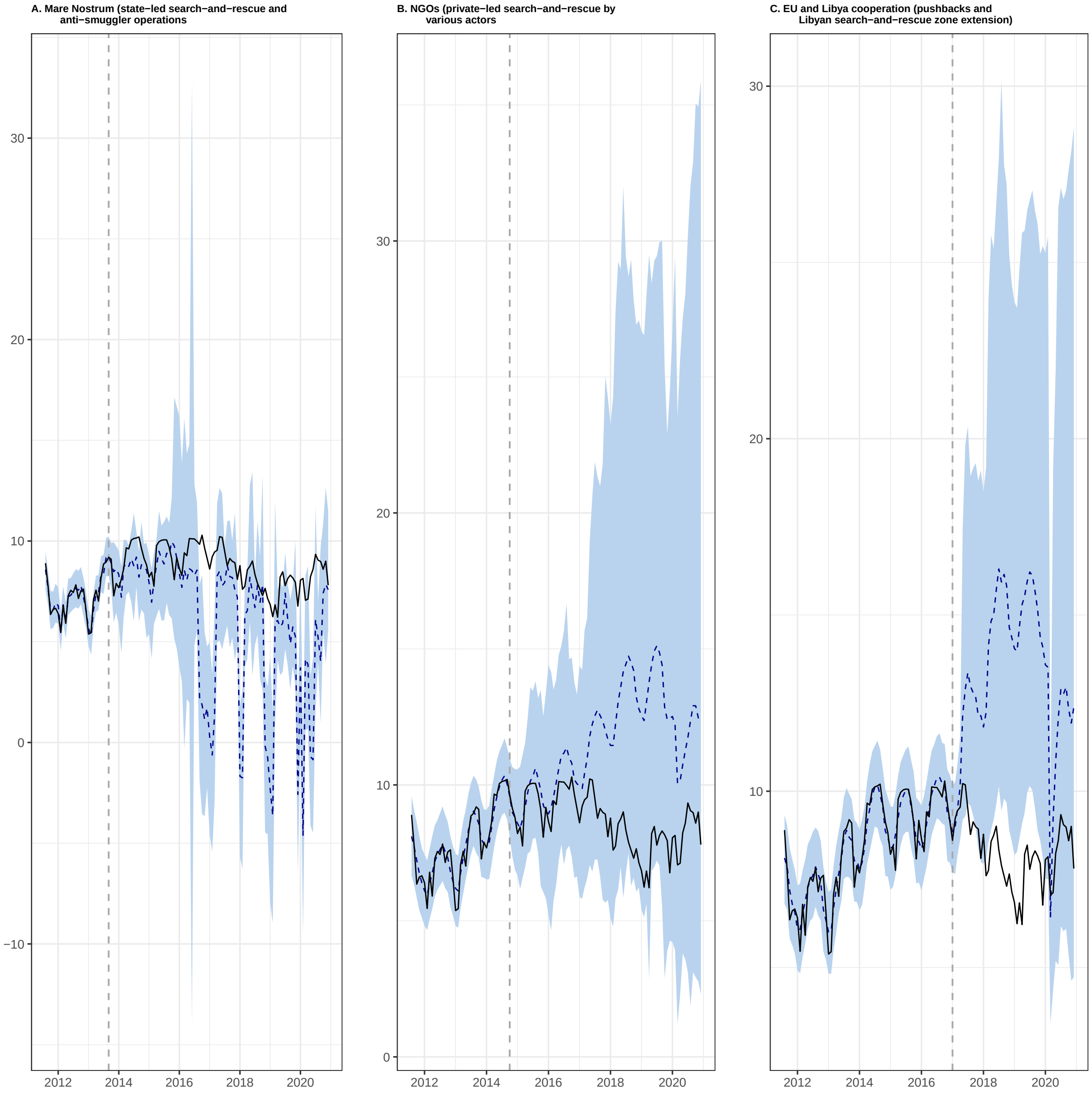

Figure S11: Results of BSTS models excluding labor market indicators for the three periods of interest

these analysis are shown in Tables S4-S6 for the three intervention periods. For each period, each model contained hundreds of variables, but only for a few of these control series we were able to detect statistically significant associations with the rejection of the reverse causal hypothesis. Given that it is not plausible that the interventions have affected the behavior of these control series, we interpret these few significant values as spurious correlations.

Table S4: Granger causality test p-values for the effect of Mare Nostrum on control series and reverse causality

control	Intervention_on_X	X_on_invervention
num_riots_Equatorial.Guinea_lag_04	0	1.0000000
num_riots_Gabon_lag_05	0	0.2638474

Note: Own elaboration. The test was done for 283 control series included in the BSTS for the Mare Nostrum intervention period. We only show the statistically significant correlations with a Bonferroni correction.

Table S5: Granger causality test p-values for the effect of private search-and-rescue on control series and reverse causality

control	Intervention_on_X	X_on_invervention
airflow_BGR_lag_6	1.28e-05	0.7787497
airflow_OMN_lag_6	1.87e-05	0.2950638
EGP_to_EURO_price_avg_lag_05	6.00e-07	0.9904117
num_riots_Gambia	0.00e+00	0.9999997
num_expvio_Cameroon_lag_01	2.00e-07	0.6623719
num_riots_Equatorial.Guinea_lag_01	0.00e+00	1.0000000
num_riots_Equatorial.Guinea_lag_02	0.00e+00	1.0000000
num_riots_Gambia_lag_03	0.00e+00	0.9999994

Note: Own elaboration. The test was done for 407 control series included in the BSTS for the private NGO-led search-and-rescue intervention period. We only show the statistically significant correlations with a Bonferroni correction.

Table S6: Granger causality test p-values for the effect of EU and Libya cooperation on control series and reverse causality

var_list_lcgs	Intervention_on_X	X_on_invervention
airflow_BGR_lag_6	0.0000128	0.7787497

disas_count_Botswana_lag_04	0.0001719	0.9998077

Note: Own elaboration. The test was done for 250 control series included in the BSTS for the EU and Libyan Coast Guard cooperation intervention period. We only show the statistically significant correlations with a Bonferroni correction.

Summary of previous studies

Table S7: Description of previous studies on the 'pull factor' claim

Year	Authors	Title	Time Frame	Identification Strategy	Data	Model	Result
2022	Hinsch, Bijak, and Hilton	"Towards More Realistic Models" (Chapter 8 of Towards Bayesian Model-Based Demography. Agency, Complexity and Uncertainty in Migration Studies)	2016–2019	Simulation - Agent Based Model - empirically calibrated with micro-level behavioral data on migrants' risk perception	Number of interceptions at the Tunisian and Libyan coasts, and deaths, and number of arrivals.	Agent Based Model of Risk and Rumours with Reality model	With their model, they closely approximated the development of numbers of arrivals and deaths occurred in the 2016–2019 period, which predicts arrivals and deaths close to the observed magnitudes. Deterrence (a policy driven increase in the risk of migration) reduced the number of arrivals through the CMR.
2021	Deianay, Maheshriz, and Mastrobuonix	Migrants at Sea: Unintended Consequences of Search and Rescue Operations	2009-2020	A mathematiacal model of irregular migration and smugglers' behavior to identify the effects of SAR and using rapidly varying crossing conditions (wind). The idea is to identify the effect of SAR from changes in the elasticities of attempts with respect to the crossing conditions.	High-frequency daily data on crossing attempts by country of origin and boat type (of restricted access), the insights of a novel model of smuggling, and plausibly exogenous, high-frequency variation in the physical conditions of each crossing attempt	A Poisson Quasi-ML regression with corrected standard errors for heteroskedasticity (i.e., HAC) and a fixed effect for week-by-year to capture endogeneity. An interaction between intervention period, weather, and boat type provides the main estimate.	Two effects are that smugglers sended boats in more adverse weather conditions and shifted from seaworthy boats to flimsy rafts thus increasing the risks. This in turn lead to more crossings in more dangerous conditions. Their claim is that the benefits of search-and-rescue have been captured by the smugglers

Table S7: Description of previous studies on the 'pull factor' claim *(continued)*

Year	Authors	Title	Time Frame	Identification Strategy	Data	Model	Result
2021	Amenta, Di Betta, and Fer-rara	The migrant crisis in the Mediterranean Sea: Empirical evidence on policy interventions	2011-2018	Policy shifts causal effects on the departures affecting the type of patrol regimes. This a comparison of two regimes adjusting for various characteristics that are considered endogenous.	Monthly data on departures (sum of arrivals and deaths) and an aggregate pseudo-panel data set of arrivals from various African countries with presence of a smuggling network (Glauco)	Probit regression and a two-stage least squares (2SLS), using as adjustment variables: weather conditions, political instability in Libya, oil production, and the odds of dying (i.e., ratio of number of deads over arrivals). Additionally, a Poisson hierarchical regression with two-way random intercepts.	SAR does not affect arrivals, but they do reduce the mortality rate along the CMR. Although arrivals were considerably higher in the SAR period, the increase was entirely due to various root causes of migration explored in the paper and the presence of smuggling networks. The determinants of departures from Libya are, therefore, root causes at countries of origin African. They suggest fighting human smuggling networks as an important policy and address push-and-pull factors in a piecewise manner
2021	Battison	Rescue on Stage: Border Enforcement and Public Attention in the Mediterranean	2014-2017	Use of exogenous variation in ships entering the Mediterranean through the Suez Canal (a proxy of maritime traddic)	High frequency geo-referenced data on rescues (weekly aggregated)	OLS regression of observed distance on a measure of survival frequency, employing fixed effects for year, quareter-of-the-year, and week-of-the-year fixed effects, and H.AC standard errors; and for the Suez canal maritime traffic instrument 2SLS	A negative association between rescue distance from the Libyan coast and future departures, and also affecting the safety of the journey, making the trip more risky.

Table S7: Description of previous studies on the 'pull factor' claim *(continued)*

Year	Authors	Title	Time Frame	Identification Strategy	Data	Model	Result
2021	Aragno and Trasi	Search and Rescue gone adrift: a magnet for migration or a lifesaver?	2014-2019	Regression with adjustment for covariates	Monthly information on departures, arrivals, deaths, proxies for economic and political stability in Libya, sea conditions, and SAR operations	Authors present a linear regression model applied to a monthly departures time series. They further estimated the risk of fatality at sea	Their main result is that proactive SAR operations do not contribute to increasing departures, though they play a role in reducing the risk of migrant deaths.
2019	Cusumano, E., & Villa, M.	Sea rescue NGOs: A pull factor of irregular migration?	2014-2019	Regression with adjustment for covariates	UNHCR, IOM, and Italian Coast Guard	Exploratory and descriptive regression analysis of the share of migrants rescued by NGOs from the total migrant arrivals. Descriptive plots and Poisson regression adjusting for weather variables and an indicator for important political instability in Libya.	Small correlation found for NGO activities and arrivals in the 2014-2019 period, and no effect of SAR on departures on the Jan-Oct 2019 period.

Table S7: Description of previous studies on the 'pull factor' claim *(continued)*

Year	Authors	Title	Time Frame	Identification Strategy	Data	Model	Result
2018	Steinhilper and Gruijters	A Contested Crisis: Policy Narratives and Empirical Evidence on Border Deaths in the Mediterranean	2010-2016	Comparison of before and after	The Migrant Files, IOM Missing Migrants Project, and FRONTEX illegal border crossings	Authors calculate the absolute and relative mortality rates, by route and over time, and perform a descriptive statistical analysis on border deaths and arrivals before and after EU border policy measures.	Authors find no clear time trend in the overall mortality rate, and they find no evidence of a higher number of arrivals in the low search-and-rescue period than in the high period, as is expected from the "pull factor" claim.
2017	Heller and Pezzani	Blaming the rescuers	2015-2016	Case study	Official documents, official statistics on arrivals - IOM and UNHCR, and estimated deaths, qualitative interviews, photographs and maps	Describe A study of migraiton dynamics in the two years and of the factors influencing the documented shifts in smugglers' tactics	Drop in number of Syrians crossing through the CMR were due to the opening of the Balkan route in Autumn 2015, but for Africans the increasing trend observed in 2015 simply continued in 2016. The period when tactical shifts in the smugglers activities (use of rubber boats) took place - and which was caused by EU operations against smugglers - coincides with only one single NGO providing search-and-rescue. They also find and inverse correlation between the mortality rate and the number of NGO assets providing search-and-rescue in the area.

Table S7: Description of previous studies on the 'pull factor' claim *(continued)*

Year	Authors	Title	Time Frame	Identification Strategy	Data	Model	Result
2017	Arsenijevic, Manzi and Zachariah	Defending humanity at sea. Are dedicated and proactive search and rescue operations at sea a "pull factor" for migration and do they deteriorate maritime safety in the central mediterranean?	204-2016	Case Study	Official statistics	Comparison of three periods: Mare Nostrum, Only Triton, and Involvement of NGOs carrying search-and-rescue	Authors find a temporal/seasonal trend of migration with a 9-17% fluctuation per year. During the period corresponding to the presence of humanitarian vessels there was not a major increase in this fluctuation (1.6% more), therefore no evidence for pull factor for attempted sea crossings. They find during the presence of NGOs there was a decrease in adverse outcomes (i.e., maritime accidents)
2016	Heller and Pezzani	Death by rescue: The lethal effects of the EU's policies of non-assistance at sea	2011-2015	Case study	Official documents, official statistics on arrivals - IOM and UNHCR, estimated deaths, qualitative interviews, photographs and maps	Descriptive evidence and qualitative information (interviews, excerpts from official documents, video footage, etc.)	The cut back on state-led search and rescue led to an increase in the mortality risk of crossing the CMR.

Note: Own elaboration.

References

1. Dearden, K., Dionis, M. S., Black, J. & Laczko, F. Calculating "death rates" in the context of migration journeys: Focus on the central mediterranean. *Series, GMDAC Briefing* (2020).

2. Farcomeni, A. How many refugees and migrants died trying to reach europe? Joint population size and total estimation. *The Annals of Applied Statistics* **16**, 2339–2351 (2022).

3. Bai, J. & Perron, P. Computation and analysis of multiple structural change models. *Journal of applied econometrics* **18**, 1–22 (2003).

4. Abadie, A., Diamond, A. & Hainmueller, J. Synthetic control methods for comparative case studies: Estimating the effect of california's tobacco control program. *Journal of the American statistical Association* **105**, 493–505 (2010).

5. Brodersen, K. H., Gallusser, F., Koehler, J., Remy, N. & Scott, S. L. Inferring causal impact using bayesian structural time-series models. *The Annals of Applied Statistics* **9**, 247–274 (2015).
